# Genome-wide identification and functional characterization of the Magnesium Transporter (MGT) gene family and its expression patterns to different anionic magnesium stresses in *Yinshania henryi*

**DOI:** 10.1186/s12864-026-12704-z

**Published:** 2026-03-02

**Authors:** Huihui Fang, Yunyan Duan, Yuke Li, Xiaolong Huang, Jing Tang, Ximin Zhang, Yin Yi, Ming Tang

**Affiliations:** 1 School of Life Sciences, Guizhou Normal University, Guiyang, 550025 China; 2 Key Laboratory of National Forestry and Grassland Administration on Biodiversity Conservation in Karst Mountainous Areas of Southwestern China, Guizhou Normal University, Guiyang, 550025 China

**Keywords:** Magnesium transporter, *Yinshania henryi*, Subcellular localization, Magnesium salt stress, *NIPA* gene

## Abstract

**Background:**

Magnesium (Mg) is an essential macronutrient required for numerous physiological and biochemical processes in plants. Both Mg deficiency and excess can disrupt plant growth and development, affecting a wide range of biological processes. Mg uptake and distribution are primarily mediated by the magnesium transporter (MGT) family. Karst landscape soils are typically enriched in calcium and magnesium, often leading to greater Mg uptake by plants than in non-karst environments. *Yinshania henryi*, a member of the Brassicaceae family endemic to the magnesium-enriched karst areas of southwestern China, demonstrates remarkable adaptability to environments with elevated Mg levels. However, the molecular basis underlying this adaptation, particularly the functional role of *MGT* genes, remains poorly understood.

**Results:**

Here, we performed a genome-wide identification and characterization of the *YhMGT* gene family using phylogenetic analysis, RNA-seq, RT-qPCR, and subcellular localization assays. Seventeen *YhMGT* genes were identified and classified into three subfamilies (*YhMRS2*, *YhNIPA*, and *YhMMgT*), displaying conserved domain architectures but diversified gene structures and promoter elements associated with abiotic stress responses. RNA-seq and RT-qPCR analyses revealed distinct and anion-dependent transcriptional responses of *YhMGT* genes to MgCl₂ and MgSO₄ treatments, with MgCl₂ generally eliciting stronger induction at higher Mg concentrations, whereas MgSO₄ induced relatively attenuated or earlier responses. Notably, several *YhNIPA* genes exhibited contrasting expression patterns between chloride- and sulfate-associated Mg stress. Subcellular localization further demonstrated functional specialization, with *YhNIPA6* localized to chloroplasts and *YhNIPA3/YhNIPA8* targeted to the plasma membrane.

**Conclusions:**

Collectively, this study reveals that *YhMGT* genes are differentially regulated by Mg²⁺ supplied with distinct anions, highlighting an anion-dependent regulatory mechanism of magnesium transport in *Y. henryi*. These findings provide new insights into how karst plants coordinate magnesium homeostasis under high-Mg environments and offer a molecular framework for understanding Mg tolerance and ecological adaptation in karst ecosystems.

**Supplementary Information:**

The online version contains supplementary material available at 10.1186/s12864-026-12704-z.

## Background

Magnesium (Mg) is an essential macronutrient required for plant growth and development, functioning in photosynthesis, ATP utilization, and enzyme activation [1–3]. Both Mg deficiency and excess can severely disturb plant growth and metabolic equilibrium. Both Mg deficiency and excess disrupt metabolic homeostasis and impair plant performance, likely starch overaccumulation, leaf chlorosis, reduce carbon dioxide fixation efficiency, lower photosynthetic capacity, and degradation of starch and soluble sugars in leaves [4–7], underscoring the need for tight regulation of cellular Mg²⁺ levels [8, 9].

In plants, the magnesium transporter (MGT) family constitutes a major component of Mg²⁺ transport machinery and has been broadly classified into three subfamilies, *MRS2*, *NIPA*, and *MMgT*, in several model and crop species [10–12]. MRS2 proteins are characterized by conserved transmembrane helices and the Gly-Met-Asn (GMN) motif, which is critical for Mg²⁺ transport activity [13]. Homologous *MRS2* genes have since been identified in diverse plant species [14–17], indicating an evolutionarily conserved role for the *MRS2* family in plant Mg²⁺ homeostasis. In contrast, the physiological roles of *NIPA*- and *MMgT*-type transporters remain less well characterized in plants, despite evidence for their conservation and potential involvement in ion transport and growth regulation [18].

Karst landforms account for approximately 15% of the Earth’s terrestrial surface [19]. Southwestern China hosts one of the largest and most continuous karst landscapes on Earth, spanning approximately 550,000 km² [20]. In karst landscapes, the combination of shallow soils, high rock exposure, and low moisture retention creates an environment in which plants regularly encounter drought and nutrient scarcity [21]. Karst soils are typically enriched in calcium and magnesium, often leading to greater Mg uptake by plants than in non-karst environments [22]. Consequently, plant species inhabiting these regions have evolved specialized physiological and molecular strategies to tolerate, resist, or avoid such edaphic stresses while sustaining growth and reproduction [23]. However, how plants maintain Mg²⁺ balance and achieve physiological tolerance under such Mg-rich conditions remains largely unknown.

*Yinshania henryi* is a Brassicaceae species endemic to limestone regions of southwestern China and exhibits strong tolerance to high-Ca environments [24], suggesting the existence of specialized ion-regulatory mechanisms. Despite the importance of Mg²⁺ homeostasis for plant adaptation, the *MGT* gene repertoire and its regulatory responses in karst-adapted species remain largely unexplored. Here, we conducted the first genome-wide identification and systematic characterization of the *MGT* family in *Yinshania henryi*. We integrated phylogenetic analysis, gene structure and conserved motif annotation, promoter cis-element profiling, subcellular localization assays, and transcriptomic and RT-qPCR analyses under MgCl₂ and MgSO₄ treatments. This study provides a comprehensive genomic resource for *YhMGT* genes and offers mechanistic insights into Mg²⁺ transport and stress adaptation in a magnesium-rich karst environment.

## Methods

### Identification of *YhMGT* genes and analysis of protein physicochemical properties

The complete genome sequence of *Y. henryi* was retrieved from the China National Center for Bioinformation (CNCB) database (accession no. CRA019022, https://www.cncb.ac.cn/search?dbId=andq=CRA019022) [24]. AtMGT protein sequences were obtained from the TAIR database (https://www.arabidopsis.org/), and their corresponding reference alignments were downloaded from UniProt (https://www.uniprot.org/). The 20 AtMGT protein sequences were used as queries to perform BLAST searches against the *Y. henryi* genome using TBtools-II (v2.149) [25], with the following parameters: output format, BLAST XML; number of threads, 2; E-value threshold, 1e-5; maximum target sequences, 500; and maximum alignments, 250. All candidate sequences were further examined for conserved MGT domains using multiple databases. Domain verification was conducted via the InterPro platform, incorporating Pfam (E-value ≤ 1e-10 with ≥ 80% domain coverage) and Prosite (E-value ≤ 1e-3), and was further confirmed using the SMART database. Only sequences containing the characteristic MGT signature domains—MRS2 (Pfam: PF01544), NIPA (Pfam: PF05653), or MMgT (Pfam: PF10270)—were retained for subsequent analyses. The physicochemical properties of the confirmed YhMGT proteins, including molecular weight, isoelectric point, amino acid composition, and instability index, were calculated using the Protein Parameter Calculator module in TBtools-II (v2.149).

### Chromosomal localization and gene duplication analysis of *YhMGT* genes

To visualize their genomic distribution, the chromosomal positions of *YhMGT* genes were assessed using the Gene Density Profile feature within TBtools-II software (v2.149). The Gene Location Visualization function module in the same software was subsequently used to map the physical positions of these genes across chromosomes and to illustrate gene duplication patterns. This approach enabled the identification of tandemly and segmentally duplicated *YhMGT* genes within the *Y. henryi* genome.

### Analysis of conserved motifs, protein domains, and gene structures

The MEME Suite (https://meme-suite.org/meme/) was employed to identify conserved motifs in YhMGT proteins, configured to allow repeated motifs, with up to 10 motifs and a minimum width of 50 amino acids. For protein domain annotation, all 17 YhMGT protein sequences were submitted to the NCBI Conserved Domain Database (CDD) (https://www.ncbi.nlm.nih.gov/Structure/cdd/cdd.shtml), and the resulting domain files were downloaded for further visualization. Exon-intron organization of *YhMGT* genes was obtained from the *Y. henryi* genome data, and the Gene Structure View (Advanced) module in TBtools-II (v2.149) was used to integrate and visualize the MEME motif results, CDD domain annotations, and exon-intron structures in a unified schematic representation.

### Phylogenetic analysis of MGT proteins in multiple plant species

To investigate the evolutionary relationships within the MGT protein family, YhMGT sequences were aligned with homologous MGT proteins from *Arabidopsis* and *O. sativa*. Twenty AtMGT sequences were retrieved from the TAIR database (https://www.arabidopsis.org/), and twenty-three OsMGT sequences were obtained from the RAP-DB database (https://rapdb.dna.affrc.go.jp/). Sequence alignment and trimming were performed using MEGA 11 software (v11.0.13), and the alignment was visualized with Jalview (v2.11.5.0) [26, 27]. A phylogenetic tree was subsequently constructed using the Maximum Likelihood (ML) method under the LG model with 1,000 bootstrap replicates to evaluate nodal support, partial gap deletion, and a 70% site coverage cutoff value were used.

### Interspecific collinearity analysis of *YhMGT* genes

Genome assemblies from multiple model and karst species—*Arabidopsis*, *O. sativa*, *Fragaria vesca*, and *Medicago truncatula*—were obtained from Phytozome (https://phytozome-next.jgi.doe.gov/) to assess the evolutionary conservation and syntenic relationships of *YhMGT* genes across diverse plant lineages. Interspecific collinearity analysis was performed using the One Step MCScanX-Super Fast module in TBtools-II (v2.149). The results were visualized through the Gene Location Visualize function in the same software to display conserved syntenic relationships and homologous gene pairs between *Y. henryi* and the selected reference species.

### Analysis of promoter cis-acting elements of *YhMGT* genes

According to the genome annotation data, the GTF/GFF3 Sequence Extract function in TBtools-II (v2.149) was used to obtain the 2,000 bp upstream promoter regions of each *YhMGT* gene. These sequences were then submitted to the PlantCARE database (https://bioinformatics.psb.ugent.be/webtools/plantcare/html/) to identify cis-acting elements involved in transcriptional regulation [28].

### Prediction of secondary and tertiary structure of YhMGT proteins

Secondary structure predictions of the YhMGT proteins were conducted with the SOPMA online platform (https://npsa.lyon.inserm.fr/cgi-bin/npsa_automat.pl?page=/NPSA/npsa_sopma.html), identifying the proportion of α-helices, β-sheets, turns, and random coils [29]. To infer the three-dimensional conformation, protein tertiary structures were modeled using the SWISS-MODEL server (https://swissmodel.expasy.org/) [30].

### Plant materials and growth conditions

In 2022, wild *Y. henryi* plants were collected from karst cliff habitats in the Kuankuoshui National Nature Reserve, Suiyang County, Guizhou Province, China. The plant material was formally identified by Prof. Renbo Zhang (Zunyi Normal University), based on morphological characteristics. A corresponding voucher specimen collected from the same population and identified by the same taxonomist has been deposited in the Chinese Virtual Herbarium (CVH) and is publicly accessible. The voucher specimen number is ZY0000995. Mature seeds were obtained from these plants and germinated under controlled greenhouse conditions at 22 °C with a 16 h light / 8 h dark photoperiod. Before sowing, seeds were pretreated at 4 °C for one week to break dormancy. They were then planted in plastic pots (8 cm height × 10 cm diameter) filled with Pindstrup nutrient soil (Ryomgaard, Denmark). Following germination, seedlings were grown in nutrient soil supplemented with half-strength Murashige and Skoog (1/2 MS) solution (Duchefa Biochemie, Haarlem, Netherlands) to promote uniform growth before experimental treatments.

### Treatment with different anionic Mg²⁺ concentrations and RNA-seq analysis

Two-month-old *Y. henryi* seedlings were treated with different concentrations of MgCl₂ and MgSO₄. Seedlings grown in 1/2 Murashige and Skoog (MS) solution containing 1.5 mM Mg²⁺, according to the reagent instructions, were used as the control group (CK). Treatment media were prepared by supplementing the 1/2 MS solution with 50, 100, 200, or 300 mM MgCl₂ or MgSO₄. Each treatment comprised three independent biological replicates. Based on previous studies [31] and preliminary phenotypic observations, distinct stress responses were observed after 14 days of treatment. Therefore, leaf tissues were harvested at this time point and immediately frozen in liquid nitrogen. High-throughput RNA sequencing was conducted by Wuhan Frasergen Gene Information Co., Ltd. using the Illumina HiSeq 4000 platform. The RNA-seq data generated in this study have been deposited in the NCBI GeneBank database under accession no. PRJNA1172955 (https://www.ncbi.nlm.nih.gov/sra/?term=PRJNA1172955).

Raw reads were quality-filtered using SOAPnuke software (v2.1.0) [32] with the following parameters: lowQual = 20, nRate = 0.005, and qualRate = 0.5, while other parameters were set to default values. Reads containing adapter sequences, those with an undetermined base (N) ratio exceeding 0.5%, and low-quality reads (in which bases with Qphred ≤ 20 accounted for more than 50% of the read length) were removed. After filtering, clean read statistics—including the number of clean read pairs, total clean bases, read length, Q20, Q30, and GC content—were summarized. The high-quality reads were first aligned to the *Y. henryi* reference genome generated in this study using HISAT2 (v2.2.1) [33], with an expected mapping rate exceeding 70% in the absence of contamination. Subsequently, Clean reads were mapped to reference transcript sequences using Bowtie2 (v2.3.5) [34]. Gene and transcript expression levels were quantified using RSEM (v1.3.3) [35] and normalized as fragments per kilobase of transcript per million mapped reads (FPKM). Differential expression analysis was conducted using DESeq2 (v1.22.2) [36] based on gene-level raw read counts from RNA-seq data. Genes with |log₂ Fold Change| ≥ 2 and adjusted P-value (padj) < 0.05 were defined as differentially expressed genes (DEGs). To account for multiple hypothesis testing, *P*-values were corrected using the Benjamini-Hochberg false discovery rate (FDR) method, and padj was used as the primary criterion for DEG identification. Expression heatmaps were generated using the Heatmap module in TBtools-II (v2.149), with hierarchical clustering applied to visualize transcriptional patterns under different Mg²⁺ treatments.

### RT-qPCR validation of *YhMGT* gene expression

To validate the RNA-seq results, quantitative real-time PCR (RT-qPCR) was performed using *Y. henryi* plants subjected to identical Mg²⁺ treatments. Total RNA was extracted from leaf tissues using an RNA Extraction Kit (LS1040, Promega, Shanghai, China) following the manufacturer’s instructions. First-strand cDNA was synthesized using the OneStep gDNA Removal and cDNA Synthesis SuperMix Kit (AE311, TransGen, Beijing, China). *YhActin1* was used as a reference gene for relative expression normalization. RT-qPCR reactions were performed in a 20 µL reaction volume containing cDNA template, SYBR Green Mix (A4004M, Biocontrast, Xiamen, China), and gene-specific primers (Table S8). Amplifications were carried out on a StepOne Real-Time PCR System (Thermo Fisher Scientific, Carlsbad, USA) under the following conditions: 95 °C for 5 min, followed by 40 cycles of 95 °C for 10 s and 60 °C for 30 s. Relative gene expression levels were calculated using the 2^⁻ΔΔCt^ method [37]. Expression data were visualized using GraphPad Prism software (v10.1.2) [38]. All RT-qPCR experiments included three independent biological replicates. Using the SPSS software (v27.0.1), statistical analyses were performed using one-way analysis of variance (ANOVA) followed by *t*-test to evaluate significant differences among treatments, with a significance threshold set at *P* < 0.05 [39]. The letters above the bars indicate the results of these comparisons: different letters represent statistically significant differences, whereas shared letters indicate no significant difference.

### Measurement of physiological indicators

To investigate the impact of varying anionic Mg²⁺ concentrations on *Y. henryi*, multiple physiological indicators—including Catalase (CAT), Superoxide Dismutase (SOD), Peroxidase (POD), Malondialdehyde (MDA), Proline (Pro), Reduced Glutathione (GSH), and Superoxide Anion—were measured. The assay kits (BC0200, BC0170, BC0090, BC0020, BC0290, BC1290, and BC1170; Solarbio, Beijing, China) were used according to the manufacturers’ protocols. All measurements included three independent biological replicates, and the *t*-test and one-way ANOVA were statistically analyzed using the SPSS software (v27.0.1), with the significance threshold set at *P* < 0.05. The letters above the bars indicate the results of these comparisons: different letters represent statistically significant differences, whereas shared letters indicate no significant difference.

### Prediction of subcellular localization and protoplast transient expression

To infer the potential intracellular distribution of YhMGT proteins, predictions were performed using the CELLO web tool (v.2.5, https://cello.life.nctu.edu.tw/) [40]. Isolation and transient expression of *Y. henryi* protoplasts were performed following previously established methods [41]. In brief, 5 g of two-month-old leaves were cut, enzymatically digested with cellulase RS-10 (MX7353, Yakult Pharmaceutical, Tokyo, Japan) and macerozyme R-10 (MX7351, Yakult Pharmaceutical, Tokyo, Japan), filtered, and washed to obtain protoplasts. Protoplasts were resuspended in MMG solution (0.4 M mannitol, 15 mM MgCl₂, 4 mM MES, pH 5.7) and transfected using polyethylene glycol (PEG). After 16 h incubation in the dark at 22 °C, subcellular localization was analyzed. Coding sequences of *YhNIPA3*, *YhNIPA6*, and *YhNIPA8* genes were cloned into the pCambia-1301-EGFP vector via *Sma*I sites (R0141V, NEB, USA). Chloroplast and plasma membrane markers (*AtRBSC* and *AtPIP2A* genes) were cloned into pCambia-1300-mCherry via *BamH*I-*Sca*I sites (R3136V and R3122V, NEB, USA). Images were acquired on an LSM 710 confocal microscope (Carl Zeiss, Jena, Germany). EGFP was excited at 488 nm with emission captured at 550–600 nm, while mCherry was excited at 514 nm and emission recorded at 588–656 nm.

## Results

### Genome-wide identification, genome mapping, and gene duplication of *YhMGT* genes

**Fig. 1:**
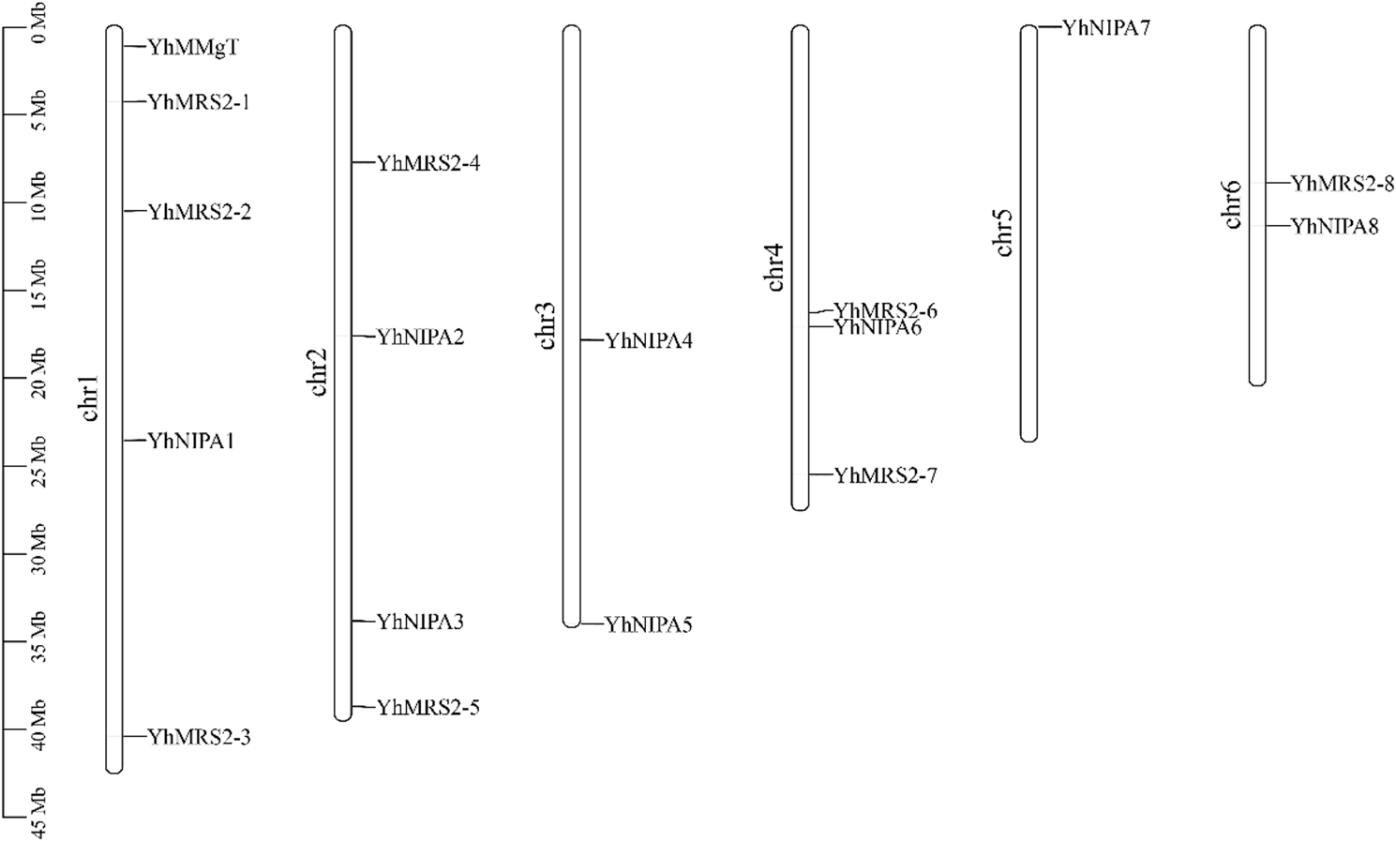
Genomic locations of the 17 *YhMGT* genes across *Yinshania henryi* chromosomes. Chromosome numbers and their lengths (Mb) are indicated on the left. Detailed gene loci are summarized in Table S2

To identify putative MGT proteins in *Y. henryi*, sequences from the *Arabidopsis MGT* subfamilies *AtMRS2*, *AtDUF803* (*NIPA*), and *AtMMgT* were used as queries to screen the *Y. henryi* genome database comprehensively. To verify these candidates, each sequence was cross-checked with homologous entries in the UniProt database. Functional domain confirmation was further carried out using Pfam, Prosite, and SMART databases. Finally, 17 YhMGT family members were identified from the *Y. henryi* genome (Table S1), consisting of eight *YhMRS2* genes, eight *YhNIPA* genes, and a single *YhMMgT* gene. The chromosomal mapping of these genes was performed using the TBtools-II software. The *YhMGT* genes were mapped to chromosomes 1 through 6. This distribution pattern confirms that the *YhMGT* family is unevenly dispersed across the genome, with a predominance on chromosomes 1 and 2 (Fig. 1).

**Fig. 2:**
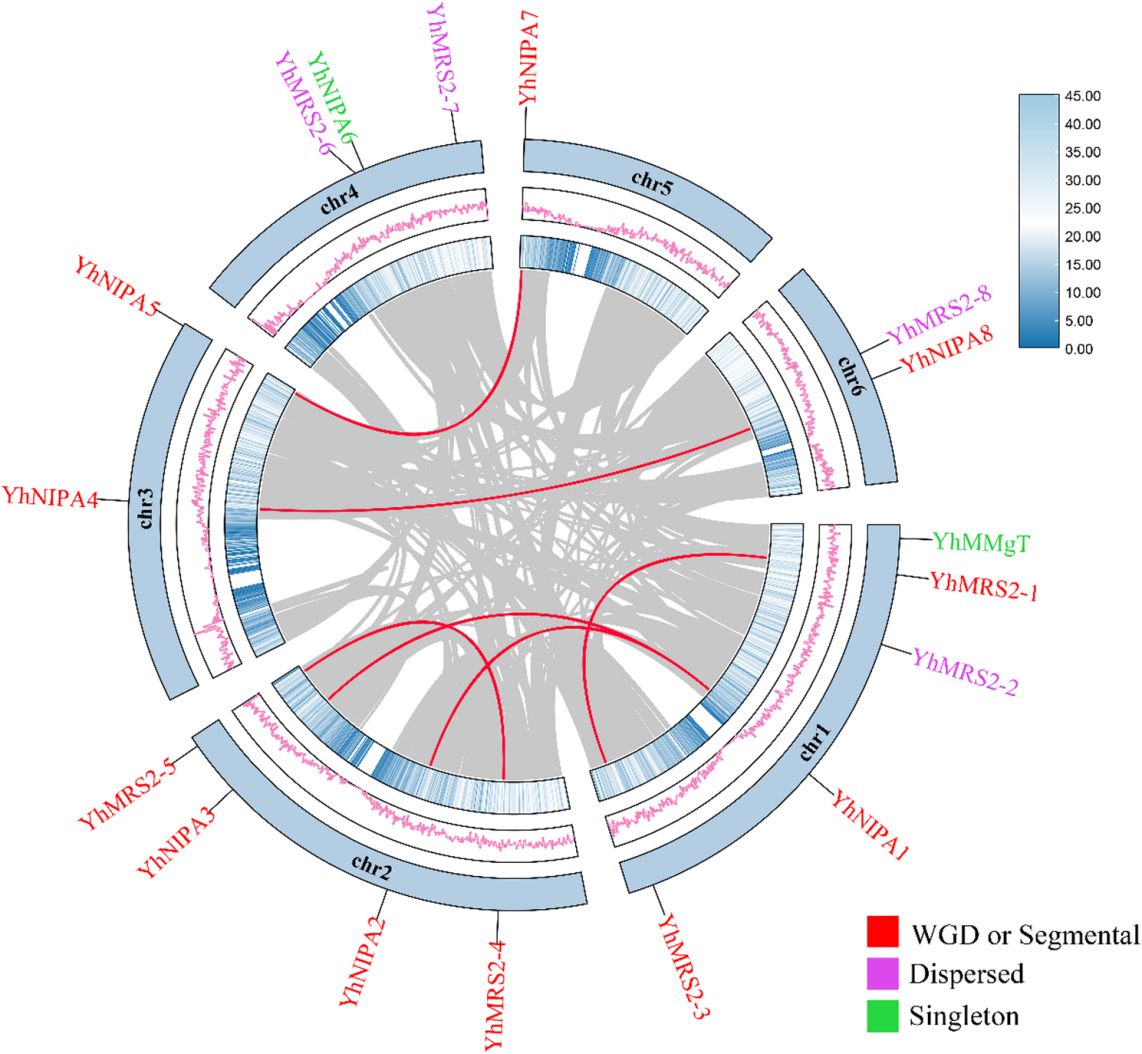
Genomic distribution and replication mechanisms of the *YhMGT* genes. Chromosomal color bands and lines corresponded to gene density. *YhMGT* genes were labeled at their chromosomal positions. Gray background lines indicat collinear blocks, and red inter-chromosomal lines connect segmentally duplicated gene pairs. Replication types were color-coded: red (WGD or Segmental), purple (Dispersed), and green (Singleton). Detailed replication data were summarized in Table S3

To investigate the evolutionary dynamics of the *YhMGT* genes, a genome-wide duplication analysis was conducted using TBtools-II software, which generated a duplication relationship map of all 17 *YhMGT* loci across the six chromosomes. As illustrated in Fig. 2, four of the *YhMGT* members were identified as dispersed duplicates located on different chromosomes (Chr1-6), whereas eleven genes formed distinct segmental or large-fragment duplication pairs spanning multiple chromosomes. In contrast, two genes—*YhNIPA6* (on Chr4) and *YhMMgT* (on Chr1)—did not show any detectable duplication relationship. These results suggested that segmental duplication events had largely contributed to both the expansion and functional differentiation of *YhMGT* genes.

### Analysis of the physicochemical characteristics of YhMGT proteins

The primary physicochemical properties of the YhMGT proteins were calculated from their amino acid sequences, and the detailed results are presented in Table 1. The lengths of the 17 YhMGT proteins varied substantially, ranging from 104 to 483 amino acids. Among them, YhMRS2-8 had the longest peptide chain (483 residues), whereas YhMMgT was the shortest (104 residues). The calculated molecular weights of these proteins ranged from 11.75 to 53.48 kDa. Isoelectric point (pI) analysis showed that most YhNIPA members had relatively high pI values between 7.66 and 9.12, except for YhNIPA5 and YhNIPA7. These pI values were higher than the corresponding grand average of hydropathicity (GRAVY) values, while the other YhMGT proteins generally displayed lower pI values. Alignment of multiple sequences showed that YhMGT proteins are highly similar to the AtMGT counterparts (Fig. S1).

**Table 1:** Properties of the 17 identified YhMGT proteins

Protein name	Amino Acid number	Molecular Weight	pI	Instability Index	Aliphatic Index	Grand Average of Hydropathicity
YhMRS2-1	256	28.96	4.7	31.62	101.33	-0.055
YhMRS2-2	460	51.28	5.51	60.36	95.63	-0.171
YhMRS2-3	401	45.00	4.87	39.41	96.78	-0.27
YhMRS2-4	443	50.47	5.19	56.27	101.87	-0.12
YhMRS2-5	445	50.59	5.27	59.28	103.15	-0.116
YhMRS2-6	440	49.45	4.96	41.50	103.89	-0.105
YhMRS2-7	436	48.35	5.26	43.41	99.54	-0.136
YhMRS2-8	483	53.48	5.02	46.44	87.47	-0.422
YhNIPA1	386	42.06	7.66	43.54	116.94	0.463
YhNIPA2	368	39.84	8.16	39.1	117.09	0.577
YhNIPA3	343	37.46	8.72	40.43	116.24	0.592
YhNIPA4	336	36.60	8.45	35.82	108.81	0.643
YhNIPA5	327	35.88	5.42	30.17	112.39	0.655
YhNIPA6	442	48.76	8.13	37.46	106.97	0.49
YhNIPA7	328	35.46	6.07	32.34	114.18	0.637
YhNIPA8	335	36.28	9.12	29.39	112.90	0.684
YhMMgT	104	11.75	5.83	28.61	100.29	0.353

### Phylogenetic analysis of YhMGT proteins

**Fig. 3:**
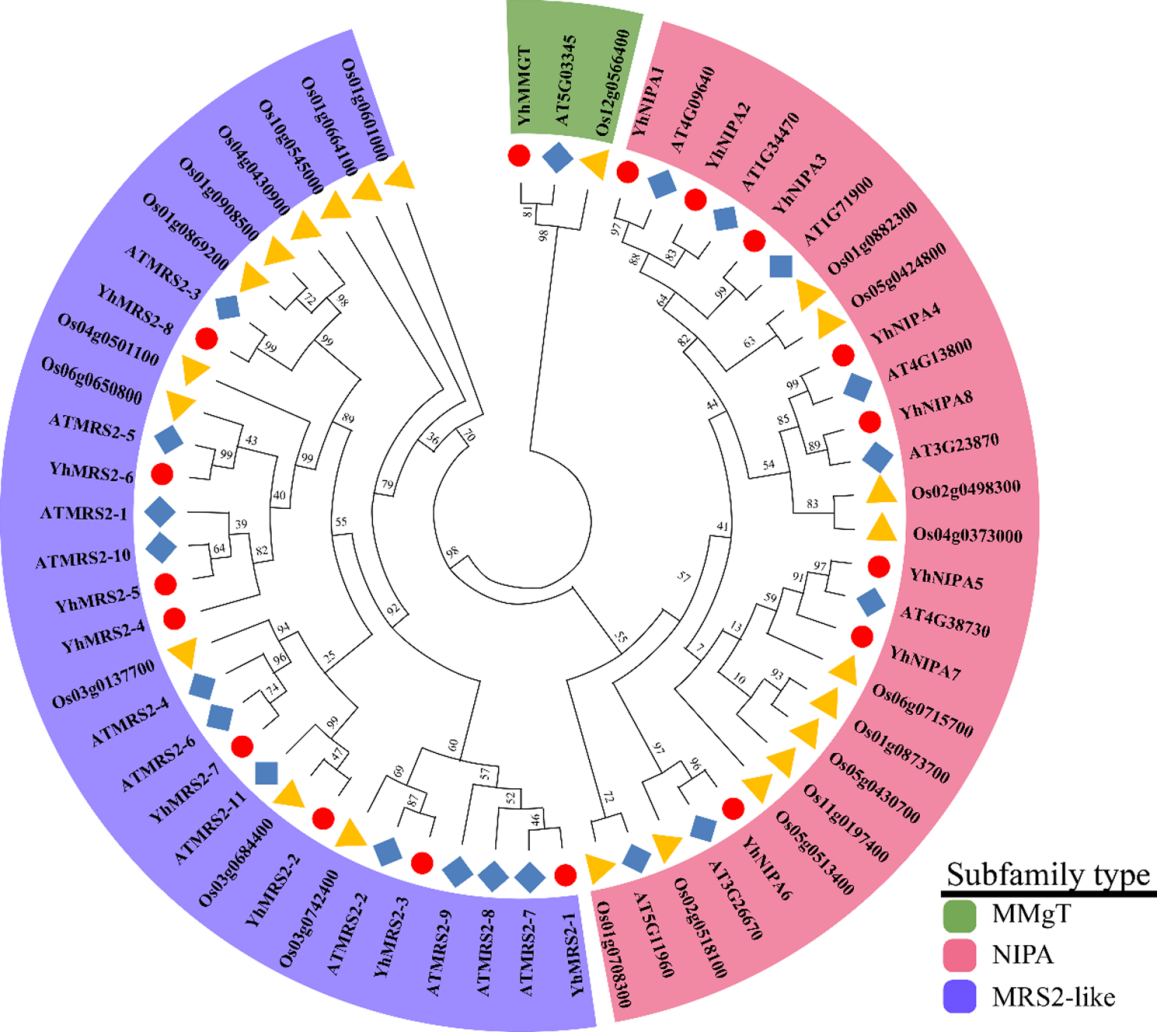
Evolutionary relationships among 60 MGT proteins from *Y. henryi*, *A. thaliana*, and *O. sativa*. The proteins were grouped into three subfamilies: *MMgT* (green), *NIPA* (red), and *MRS2* (blue). Proteins from each species are denoted by specific symbols: yellow triangles for OsMGTs from *O. sativa*, blue rhombuses for AtMGTs from *A. thaliana*, and red circles for YhMGTs from *Y. henryi*. The numbers on the nodes represent branch support values. Detailed information was summarized in Table S5

The evolutionary relationships of MGT proteins from multiple plant species were examined using a phylogenetic analysis based on the Maximum Likelihood (ML) method. A total of 60 MGT protein sequences were incorporated in the analysis, comprising 17 YhMGTs, 20 AtMGTs, and 23 OsMGTs. According to the conserved structural features of the protein sequences and previously reported classifications of MGT family members, the 17 YhMGT proteins were grouped into three well-defined clades, designated as the *MRS2*, *NIPA*, and *MMgT* subgroups (Fig. 3). Overall, the phylogenetic organization demonstrated a high degree of conservation and structural correspondence of MGT family members among *Y. henryi*, *A. thaliana*, and *O. sativa*, suggesting that these genes shared similar evolutionary trajectories across species.

### Conserved motif and exon-intron structure analysis of *YhMGT*

**Fig. 4:**
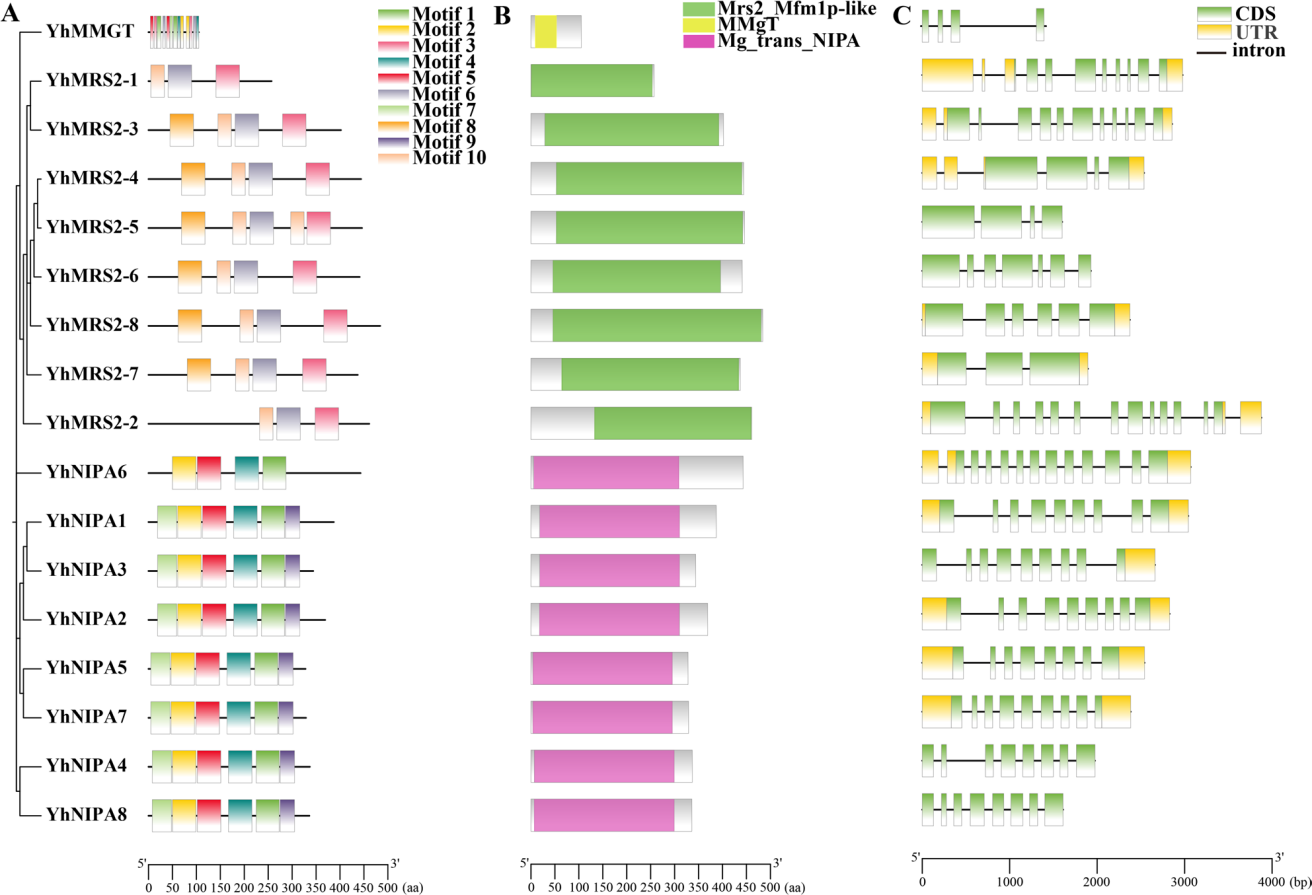
Analysis of the characteristics of the *YhMGT* family genes. **A** Phylogenetic relationships and conserved motif distribution of the 17 YhMGT proteins. The branch lengths of the phylogenetic tree reflect sequence divergence. Ten distinct motifs are color-coded. Protein representations are scaled according to their actual sequence lengths. **B** Conserved domain architecture of YhMGT proteins. The Mrs2_Mfmlp-like, MMgT, and Mg_trans_NIPA domains are depicted by green, yellow, and purple boxes, respectively. The schematic length of each protein is proportional to its actual length. **C** Gene structure of *YhMGT* members. Untranslated regions (UTRs) and exons are represented by yellow and green boxes, respectively, with black lines indicating introns. The schematic length of each gene is proportional to its actual length

The MEME online platform was employed to detect conserved motifs across the 17 YhMGT protein sequences, providing insights into their structural and functional variation. A total of ten distinct motifs were detected (Fig. 4A). In general, proteins with higher sequence homology displayed more similar motif compositions and arrangements. However, considerable variation was observed among individual YhMGT members. In detail, motifs 3, 6, 8 and 10 were present in six YhMRS2 proteins; YhMRS2-1 and YhMRS2-2 lack motif 8 compared with other YhMRS2 members. Motifs 1, 2, 4, 5, 7, and 9 were detected in the eight YhNIPA proteins; notably, YhNIPA6 lacked motifs 7 and 9 compared with other YhNIPA members; The YhMMgT protein contained motifs 1 through 7. Comprehensive details of the identified motifs are provided in Table S4. In general, the structural features of YhMGT proteins were relatively well conserved. Domain prediction confirmed that YhMRS2 proteins contained the Mrs2_Mfmlp-like domain, YhNIPA proteins harbored the Mg_trans_NIPA domain, and YhMMgT possessed the MMgT domain (Fig. 4B). Structural analysis indicated that the 17 *YhMGT* genes exhibit diverse patterns of exon and intron organization. Exon counts within the CDS regions varied among *YhMGT* genes, ranging from three in *YhMRS2-7* to thirteen in *YhMRS2-2*. On average, *MRS2*-like genes contained three to thirteen exons, *NIPA* subgroup genes had seven to twelve, and *MMgT* comprised four exons (Fig. 4C). Such differences in exon composition may reflect potential functional diversification among the three subgroups. In contrast, the conservation of motif patterns within each group suggested close evolutionary relationships.

### Analysis of interspecies collinearity of *MGT* genes between *Y. henryi* and selected species

**Fig. 5:**
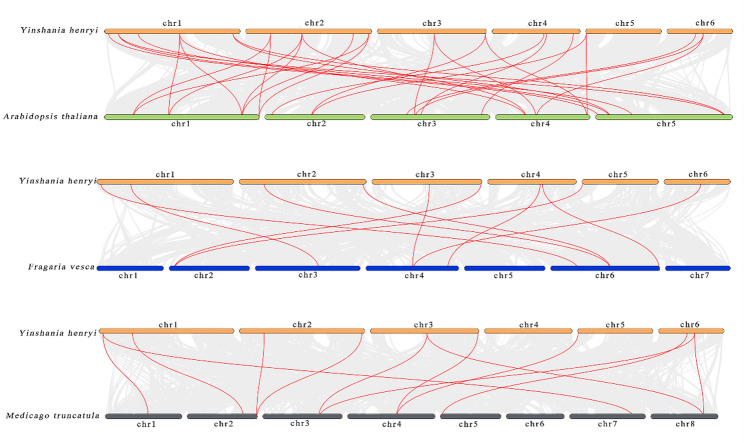
The syntenic relationships of *MGT* genes in *Y. henryi* were compared with those in *A. thaliana*, *F. vesca*, and *M. truncatula*. Red lines highlight the conserved gene pairs between *YhMGTs* and their corresponding homologs. Gray lines depict the background genomic collinearity. Horizontal bars represent chromosomes, with numbers labeled above or below

Comparative synteny mapping was carried out between *Y. henryi* and four selected species, comprising two model species (*A. thaliana* and *O. sativa*), two common Karst plants (*F. vesca*, and *M. truncatula*)—to understand the evolutionary patterns of *YhMGT* genes better. As illustrated in Fig. 5, extensive collinearity was observed between *YhMGT* genes and *A. thaliana* homologs, with 17 *YhMGT* genes showing syntenic correspondence to *AtMGT* genes. Ten and nine *YhMGT* members were found to share collinear relationships with *F. vesca* and *M. truncatula* homologs, respectively. In contrast, no collinearity was detected with *O. sativa* (Fig. S2). These results indicate that *YhMGT* genes exhibit higher evolutionary conservation and are more closely related to their counterparts in dicotyledonous species than to those in monocots. These results suggest that the majority of *YhMGT* orthologs likely originated after the evolutionary divergence of dicotyledonous and monocotyledonous lineages, as indicated by strong collinearity with other dicots.

### Prediction of secondary and tertiary structure of YhMGT proteins

The structural features of all 17 YhMGT proteins were further analyzed by predicting their secondary structures using the SOPMA online tool. As summarized in Table 2, the predicted secondary structures mainly consisted of α-helices, extended strands, and random coils. Among these, the α-helix proportion was highest in YhMRS2 proteins (52.59%–73.05%), whereas YhNIPA proteins displayed the lowest α-helix content (37.56%–48.78%). The random coil fraction was lowest in YhMMgT protein (31.73%). In contrast, YhNIPA proteins showed the highest proportion of extended strands (12.22%–18.90%), while YhMRS2 proteins contained the least (2.73%–8.48%). Notably, no β-turn structures were detected in any of the 17 YhMGT proteins. The tertiary structures of YhMGT proteins were further predicted, and the results are presented in Fig. S3. The YhMRS2 and YhMMgT proteins each contained two transmembrane helices, whereas the YhNIPA proteins possessed nine transmembrane helices. These findings indicated that YhNIPA proteins, despite their relatively low α-helix content, exhibited a more complex transmembrane topology compared with YhMRS2 and YhMMgT. Overall, these structural characteristics suggest that YhMGT proteins may regulate Mg²⁺ homeostasis across cellular membranes, maintaining magnesium ion balance within plant cells.

**Table 2:** Secondary structure of YhMGT proteins

Protein Names	α-helix (%)	extended strand (%)	Random coil (%)
YhMRS2-1	73.05%	24.22%	2.73%
YhMRS2-2	55.00%	36.52%	8.48%
YhMRS2-3	58.10%	35.66%	6.23%
YhMRS2-4	53.95%	40.41%	5.64%
YhMRS2-5	53.03%	40.22%	6.74%
YhMRS2-6	55.45%	37.95%	6.59%
YhMRS2-7	53.90%	40.14%	5.96%
YhMRS2-8	52.59%	41.82%	5.59%
YhNIPA1	41.97%	42.75%	15.28%
YhNIPA2	39.95%	41.58%	18.48%
YhNIPA3	45.77%	37.03%	17.20%
YhNIPA4	47.32%	34.23%	18.45%
YhNIPA5	47.71%	33.64%	18.65%
YhNIPA6	37.56%	50.23%	12.22%
YhNIPA7	48.78%	32.32%	18.90%
YhNIPA8	45.37%	35.82%	18.81%
YhMMgT	57.69%	31.73%	10.58%

### Identification and analysis of promoter cis-acting elements of *YhMGT* genes

**Fig. 6:**
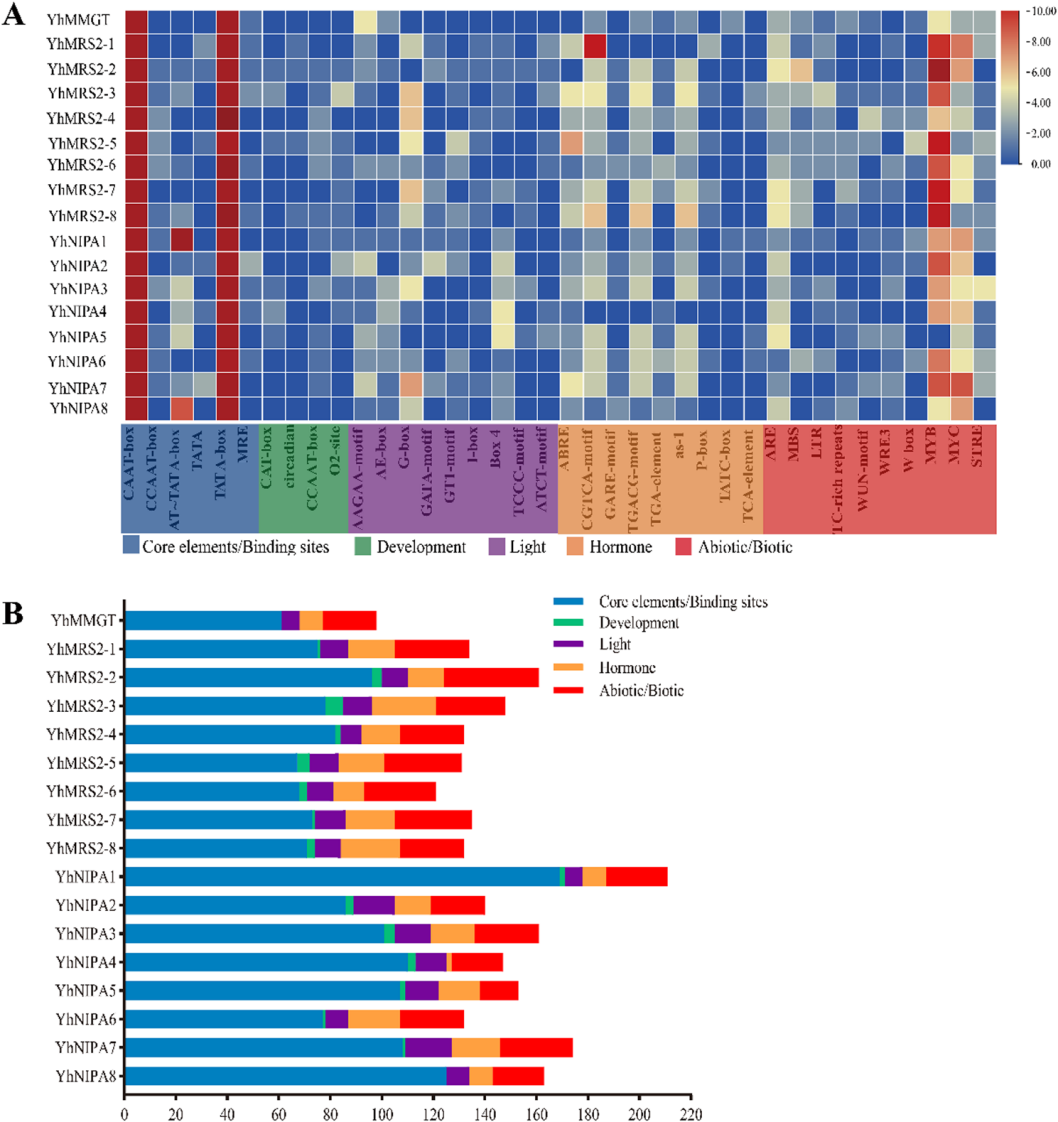
Cis-acting elements analysis in the promoters of 17 *YhMGT* genes. **A** Distribution of identified elements. The diagram displays 38 cis-acting elements, belonging to five types, identified within the 2 kb promoter regions. The color scale indicates the count of each element in different genes. **B** The total number of cis-acting elements across the five functional categories was summarized. The graph summarizes the total number of the five types for each *YhMGT* gene. See Table S6 for details

To explore the potential regulatory functions of *YhMGT* genes, 2,000 bp upstream promoter regions of the 17 *YhMGT* coding sequences were extracted and analyzed using the PlantCARE online tool. As shown in Fig. 6, the promoters of all *YhMGT* genes harbored multiple core promoter motifs (Fig. 6A, B). The promoters of *YhMGT* genes contained a diverse array of hormone-responsive motifs, among which ABRE was the most abundant. Each *YhMGT* gene harbored at least two hormone-responsive motifs, suggesting involvement in multiple hormone signaling pathways. Additionally, light-responsive elements were widely distributed, with the G-box appearing most frequently. Most *YhMGT* gene promoters were enriched for ARE and drought-responsive motifs, including MBS, MYB, and MYC, and all genes contained LTR elements. Defense- and wound-related motifs, including WUN and WRE3, were also detected, supporting a potential role for *YhMGT* genes in hormone-regulated pathways that enhance growth, development, and resistance to environmental stresses. Comparison of cis-actin elements among the 17 genes revealed no significant differences in the total number of elements or in the number of stress-related motifs. This finding indicates that the diverse expression patterns of *YhMGT* genes were likely governed by complex and finely tuned regulatory networks rather than by variations in promoter structure alone.

### RNA-seq analysis with different anionic magnesium salt stress

Furthermore, we explored the expression patterns of all of the *YhMGT* genes. Seedlings of *Y. henryi*, aged two months and propagated several generations of self-pollination, were maintained in a controlled greenhouse and treated with MgCl₂ and MgSO₄ at 50 mM, 100 mM, 200 mM, and 300 mM concentrations for 14 days. Seedlings grown in 1/2 MS solution served as controls (CK). Exposure to high Mg²⁺ concentrations (300 mM MgCl₂ and MgSO₄) induced leaf yellowing and withering, whereas treatment at 100 mM did not result in observable phenotypic changes compared with CK. Leaf tissues were collected for RNA-seq analysis, and 27 plant samples were extracted and sequenced, producing 320 GB paired-end sequencing data. In total, 31.09–47.89 million clean-read pairs were obtained from each sample. All samples had Q30 values greater than 91.47%, and the GC content ranged from 45.13% to 46.95% (Table 3).

**Table 3:** RNA-seq sample information and sequencing statistics

Sample	Clean Reads Pairs	Clean base (bp)	Length (bp)	Q20 (%)	Q30 (%)	GC content (%)
CK_1	38,117,672	11,435,301,600	150;150	98.06;96.70	95.11;92.78	46.73;46.58
CK_2	37,263,875	11,179,162,500	150;150	98.68;97.63	96.40;94.13	46.82;46.77
CK_3	32,728,105	9,818,431,500	150;150	97.97;97.21	94.90;93.83	46.83;46.71
MC50_1	44,543,004	13,362,901,200	150;150	97.89;97.06	94.70;93.55	46.23;46.09
MC50_2	32,600,979	9,780,293,700	150;150	98.21;97.12	95.44;93.69	46.07;45.96
MC50_3	38,087,163	11,426,148,900	150;150	98.29;97.52	95.47;94.09	46.68;46.59
MC100_1	31,090,545	9,327,163,500	150;150	97.60;95.99	94.16;91.47	45.90;45.66
MC100_2	35,783,578	10,735,073,400	150;150	98.24;97.35	95.51;94.16	46.30;46.19
MC100_3	34,080,459	10,224,137,700	150;150	98.09;97.22	95.14;93.83	46.51;46.41
MC200_1	36,116,870	10,835,061,000	150;150	98.38;96.98	95.75;92.97	45.29;45.13
MC200_2	39,586,269	11,875,880,700	150;150	98.22;97.21	95.47;93.87	45.99;45.85
MC200_3	37,575,713	11,272,713,900	150;150	98.60;97.38	96.26;93.78	45.95;45.85
MC300_1	38,392,180	11,517,654,000	150;150	98.46;97.41	95.90;93.92	45.28;45.15
MC300_2	38,754,066	11,626,219,800	150;150	98.28;97.38	95.41;93.77	45.32;45.19
MC300_3	38,416,554	11,524,966,200	150;150	98.19;97.55	95.40;94.58	45.52;45.38
MS50_1	31,569,062	9,470,718,600	150;150	98.36;97.03	95.78;93.42	46.66;46.57
MS50_2	38,522,437	11,556,731,100	150;150	98.34;97.20	95.75;93.84	46.82;46.75
MS50_3	34,619,189	10,385,756,700	150;150	98.62;97.27	96.26;93.41	46.69;46.62
MS100_1	37,900,529	11,370,158,700	150;150	98.24;96.57	95.53;92.57	46.67;46.49
MS100_2	47,894,245	14,368,273,500	150;150	98.08;97.11	95.14;93.61	46.74;46.58
MS100_3	33,748,808	10,124,642,400	150;150	98.10;96.98	95.20;93.42	46.95;46.83
MS200_1	44,580,646	13,374,193,800	150;150	98.13;97.36	95.27;94.21	46.18;46.07
MS200_2	38,000,727	11,400,218,100	150;150	98.03;96.93	94.99;93.22	46.42;46.29
MS200_3	39,668,681	11,900,604,300	150;150	98.25;97.22	95.55;93.88	46.33;46.19
MS300_1	34,691,601	10,407,480,300	150;150	98.32;97.13	95.70;93.69	45.45;45.31
MS300_2	36,571,373	10,971,411,900	150;150	98.14;96.72	95.25;92.82	45.73;45.56
MS300_3	36,080,641	10,824,192,300	150;150	98.22;97.48	95.26;93.87	45.83;45.70

CK, control; MC50/100/200/300, 50, 100, 200, and 300 mM MgCl₂ treatments; MS50/100/200/300, 50, 100, 200, and 300 mM MgSO₄ treatments. The numbers following MC and MS represented biological replicates

**Fig. 7:**
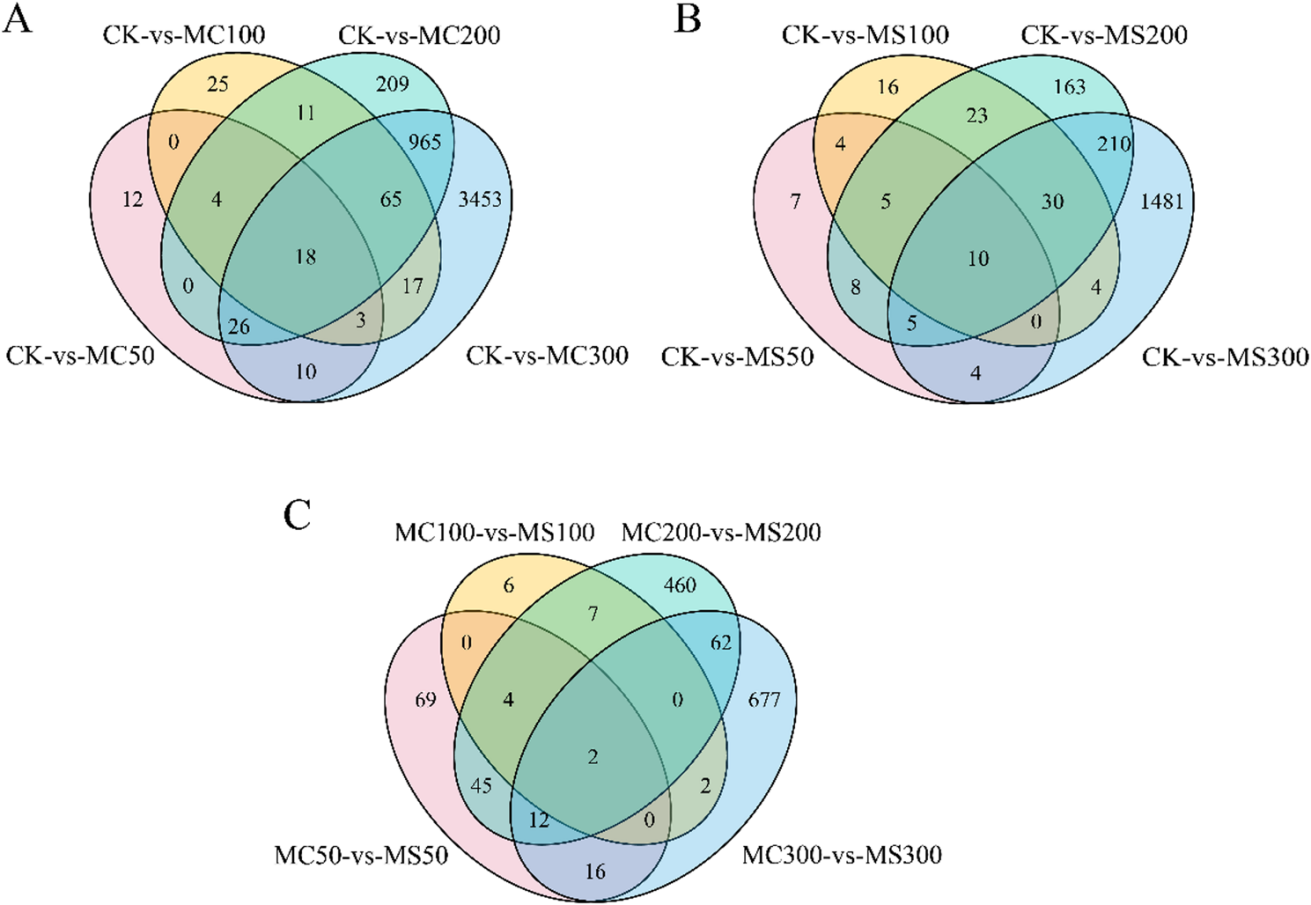
Analysis of DEGs among the different experimental conditions. **A** Comparison of CK with different concentrations of MgCl_2_ treatment. **B** Comparison of CK with different concentrations of MgSO_4_ treatment. **C** Comparison of MgCl_2_ and MgSO_4_ treatments at the same concentration. CK, control; MC50/100/200/300, 50, 100, 200, and 300 mM MgCl₂ treatments; MS50/100/200/300, 50, 100, 200, and 300 mM MgSO₄ treatments

Table S7 shows a concentration-dependent increase in DEG numbers under both MgCl₂ and MgSO₄ treatments, with the largest change observed at 300 mM. At each matched concentration, MgCl₂ induced more DEGs than MgSO₄. Venn analysis (Fig. 7) identified shared and unique DEGs across treatments, with treatment-specific DEGs becoming more abundant at higher Mg²⁺ levels.

GO and KEGG enrichment analyses were conducted to investigate the functional categories and pathways associated with DEGs induced by MgCl₂ and MgSO₄ (Tables 4 and 5). GO enrichment results (Table 4; Fig. S4) showed that DEGs from all treatment groups were mainly enriched in stress-related terms, including response to stress/stimulus/chemical. MgCl₂-responsive DEGs were additionally enriched in transcriptional regulation, whereas MgSO₄-responsive DEGs were more associated with secondary metabolism and cell wall-related functions.

**Table 4:** Significantly enriched GO terms of the DEGs generated from different treatments

GO Category	CK vs. MC100	CK vs. MC200	CK vs. MC300
Biological Process	Response to oxygen-contain compound Response to chemical Response to hormone	Response to stimulus Response to chemical Response to stress	Response to stimulus Response to stress Response to chemical
Molecular Function	Monooxygenase activity	Transcription regulator activity DNA-binding transcription factor activity Monooxygenase activity	Transcription regulator activity Oxidoreductase activity DNA-binding transcription factor activity

GO Category	CK vs. MS100	CK vs. MS200	CK vs. MS300
Biological Process	None	Response to oxygen-containing compound	Response to stimulus Response to stress Response to chemical Response to osmotic stress
Molecular Function	UDP-glucosyltransferase activity	Monooxygenase activity	Oxidoreductase activity Glycosyltransferase activity RNA binding

CK, control; MC50/100/200/300, 50, 100, 200, and 300 mM MgCl₂ treatments; MS50/100/200/300, 50, 100, 200, and 300 mM MgSO₄ treatments

**Table 5:** Significantly enriched KEGG pathways of the DEGs generated from different treatments

CK vs. MC50	CK vs. MC100	CK vs. MC200	CK vs. MC300
Cysteine and methionine metabolism Two-component system	Plant hormone signal transduction Tryptophan metabolism Flavonoid biosynthesis	Plant hormone signal transduction Plant-pathogen interaction Cell cycle	Plant hormone signal transduction Photosynthesis Porphyrin metabolism

CK vs. MS50	CK vs. MS100	CK vs. MS200	CK vs. MS300
Ribosome biogenesis in eukaryotes Pyruvate metabolism Glycine, serine and threonine metabolism	Cysteine and methionine metabolism Ether lipid metabolism Pyruvate metabolism	Plant hormone signal transduction Plant-pathogen interaction Ether lipid metabolism Cutin, suberine and wax biosynthesis	Plant hormone signal transduction Plant-pathogen interaction Starch and sucrose metabolism Photosynthesis-antenna proteins utin, suberine and wax biosynthesis

CK, control; MC50/100/200/300, 50, 100, 200, and 300 mM MgCl₂ treatments; MS50/100/200/300, 50, 100, 200, and 300 mM MgSO₄ treatments

KEGG enrichment results (Table 5; Fig. S5) showed both common and divergent pathway responses to MgCl₂ and MgSO₄. Across 100–300 mM treatments, DEGs were repeatedly enriched in Plant hormone signal transduction and Plant-pathogen interaction. Under high MgCl₂, Porphyrin metabolism was significantly enriched, whereas high MgSO₄ preferentially enriched Cutin, suberine and wax biosynthesis, indicating anion-dependent differences in stress-responsive pathways.

**Fig. 8:**
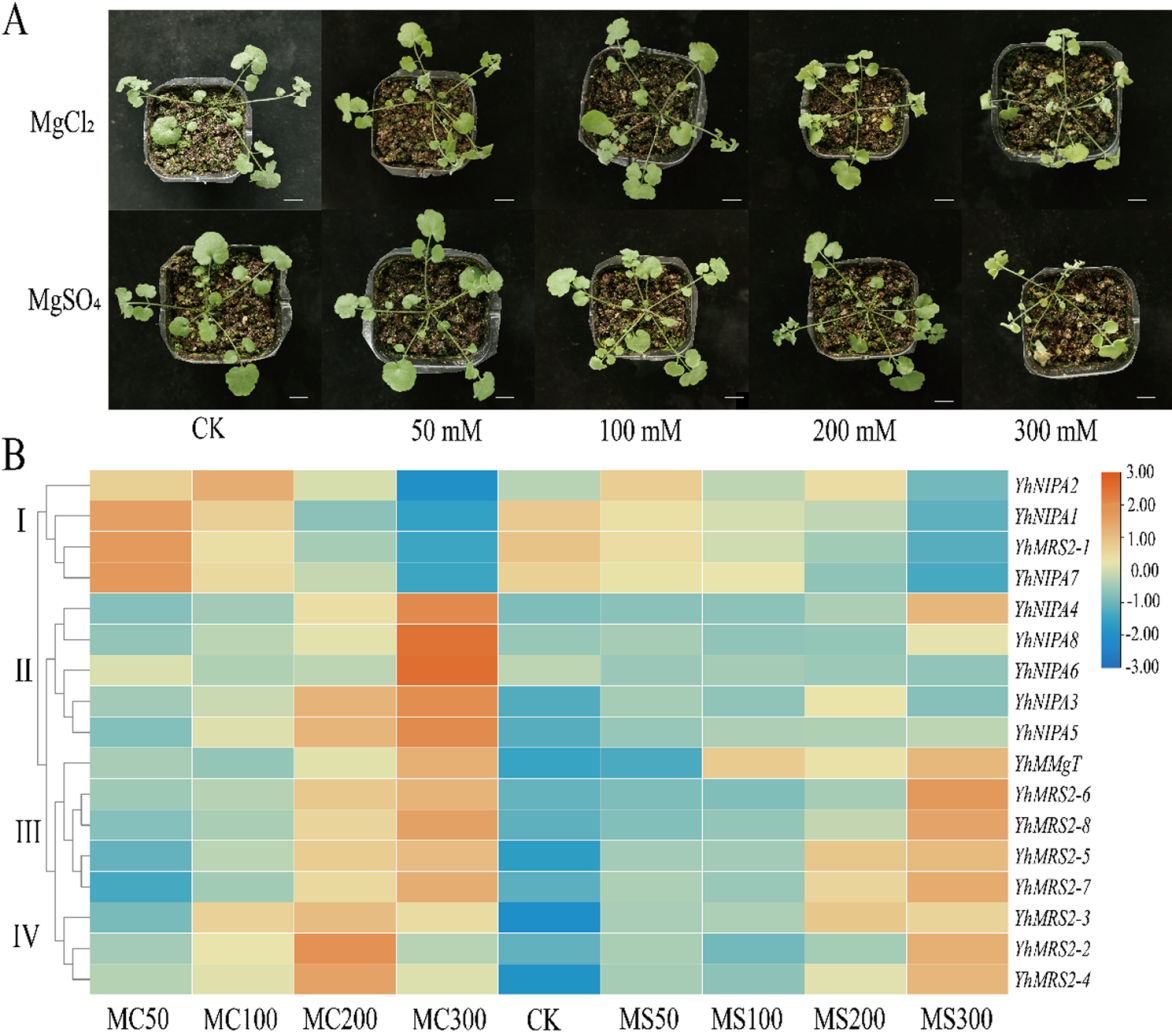
RNA-seq analysis of *YhMGT* gene expression under MgCl₂ and MgSO₄ treatments. **A** Phenotypes of *Y. henryi* seedlings treated with different concentrations of MgCl₂ and MgSO₄. Bar = 1 cm. **B** Expression patterns of *YhMGT* gene based on RNA-seq. CK, control; MC50/100/200/300, 50, 100, 200, and 300 mM MgCl₂ treatments; MS50/100/200/300, 50, 100, 200, and 300 mM MgSO₄ treatments. Gene expression levels were quantified as log₂ (FPKM + 1) values for all treatments. These values were then normalized and analyzed by hierarchical clustering. The color scale indicates expression levels, from high (red) to low (blue)

RNA-seq analysis revealed anion-dependent transcriptional responses of *YhMGT* genes to MgCl₂ and MgSO₄ treatments (Fig. 8). Overall, MgCl₂ elicited a stronger and more dynamic transcriptional response than MgSO₄, as evidenced by a greater number of *YhMGT* genes showing pronounced induction at elevated Mg²⁺ concentrations. Under MgCl₂ stress, *YhMGT* genes could be broadly classified into two major response patterns. Group I genes exhibited a transient induction, with increased expression at lower Mg²⁺ concentrations followed by a decline at 200 and 300 mM. In contrast, Groups II, III, and IV displayed sustained upregulation, reaching maximal expression levels at 200 or 300 mM MgCl₂. Compared with MgCl₂, MgSO₄ induced overall weaker and more moderated transcriptional responses. While the expression trends of Groups I, III, and IV under MgSO₄ were generally similar to those observed under MgCl₂, Group II genes showed greater variability. Specifically, *YhNIPA5* and *YhNIPA6* exhibited relatively stable expression across increasing Mg²⁺ concentrations, whereas *YhNIPA3* reached its highest expression at 200 mM MgSO₄, indicating a more constrained transcriptional activation under sulfate-associated Mg²⁺ stress. Collectively, these results demonstrate that *YhMGT* genes are differentially regulated depending on the accompanying anion of Mg²⁺, with chloride-associated Mg²⁺ inducing broader and stronger transcriptional activation than sulfate-associated Mg²⁺, highlighting a clear anion-dependent regulatory pattern in *Y. henryi*.

### *YhMGT* gene expression analysis by RT-qPCR

**Fig. 9:**
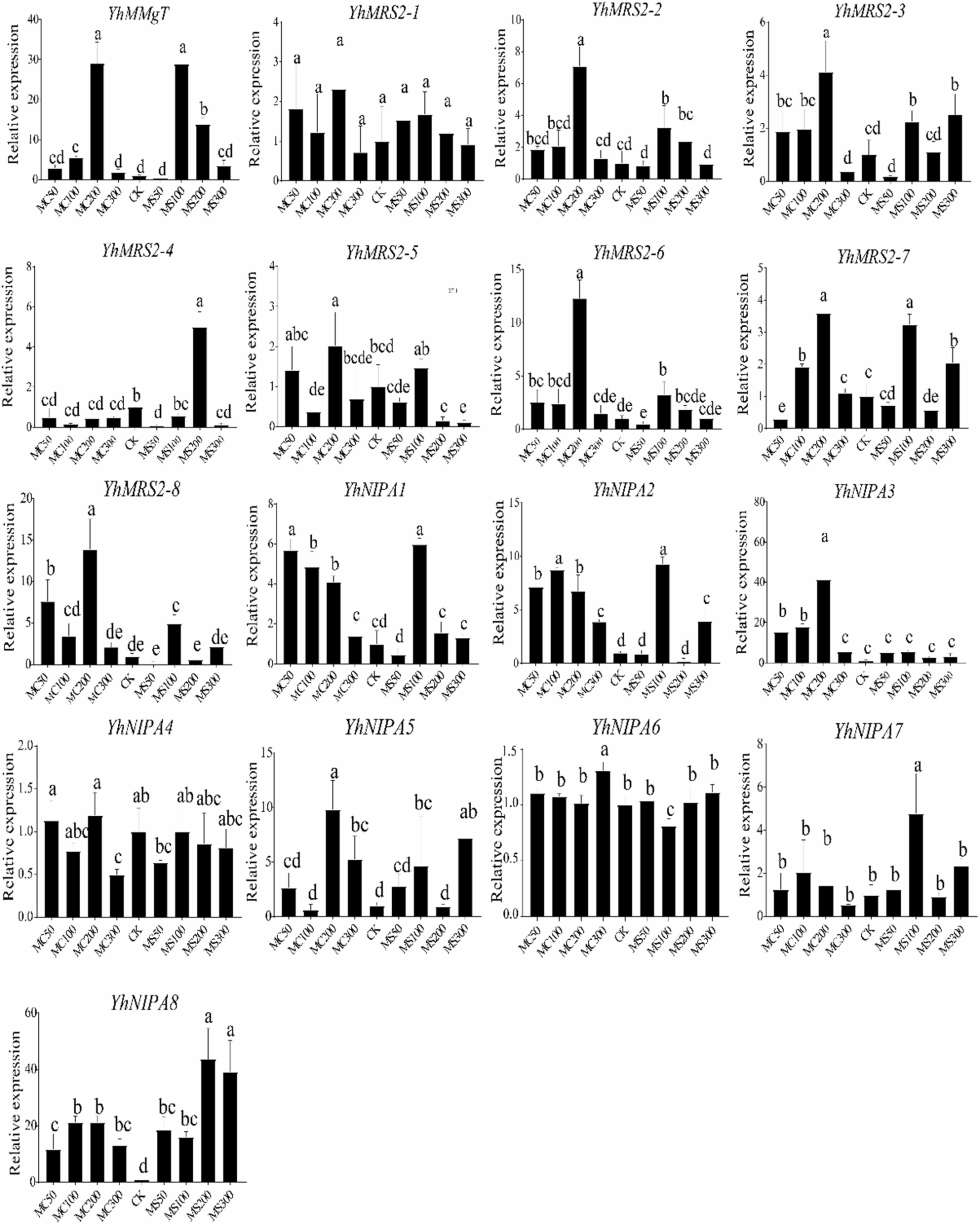
RT-qPCR expression profiling of *YhMGT* genes under MgCl₂ and MgSO₄ treatments. CK, control; MC50/100/200/300, 50, 100, 200, and 300 mM MgCl₂ treatments; MS50/100/200/300, 50, 100, 200, and 300 mM MgSO₄ treatments. Data represent mean ± SD of three biological replicates. Different letters above the bars denote statistically significant differences among treatments at the 0.05 level. Two letters indicate that the two treatments share one letter; the difference is not significant. Three letters indicate that two treatments share one letter; the difference is not significant

Quantitative real-time PCR (RT-qPCR) was performed to validate the expression patterns of representative *YhMGT* genes identified from the RNA-seq analysis. Leaf samples were collected from two-month-old *Y. henryi* seedlings subjected to CK and four Mg²⁺ treatments (50, 100, 200, and 300 mM) for 14 days. Overall, the RT-qPCR results showed expression trends largely consistent with the RNA-seq data (Fig. 9), confirming the reliability of the transcriptomic analysis and supporting the differential regulation of *YhMGT* genes under Mg²⁺ stress imposed by distinct magnesium salts. Clear anion-dependent expression differences were observed among *YhNIPA* genes. *YhNIPA3* was preferentially induced by MgCl₂, exhibiting progressive upregulation at 50, 100, and 200 mM followed by a decline at 300 mM, whereas MgSO₄ treatment did not cause significant changes in its expression across all tested concentrations (Fig. 9). In contrast, *YhNIPA8* responded to both MgCl₂ and MgSO₄, with MgSO₄ eliciting a relatively stronger induction, indicating a sulfate-associated enhancement of *YhNIPA8* transcription. *YhNIPA6* displayed a highly specific response, showing no substantial expression changes under most conditions but a distinct induction exclusively at 300 mM MgCl₂. Collectively, the RT-qPCR results demonstrate that key *YhMGT* genes exhibit distinct and anion-dependent transcriptional responses, with certain members preferentially responding to chloride-associated Mg²⁺ stress, while others show broader or sulfate-enhanced expression patterns.

### Determination of plant physiological and biochemical indicators

**Fig. 10:**
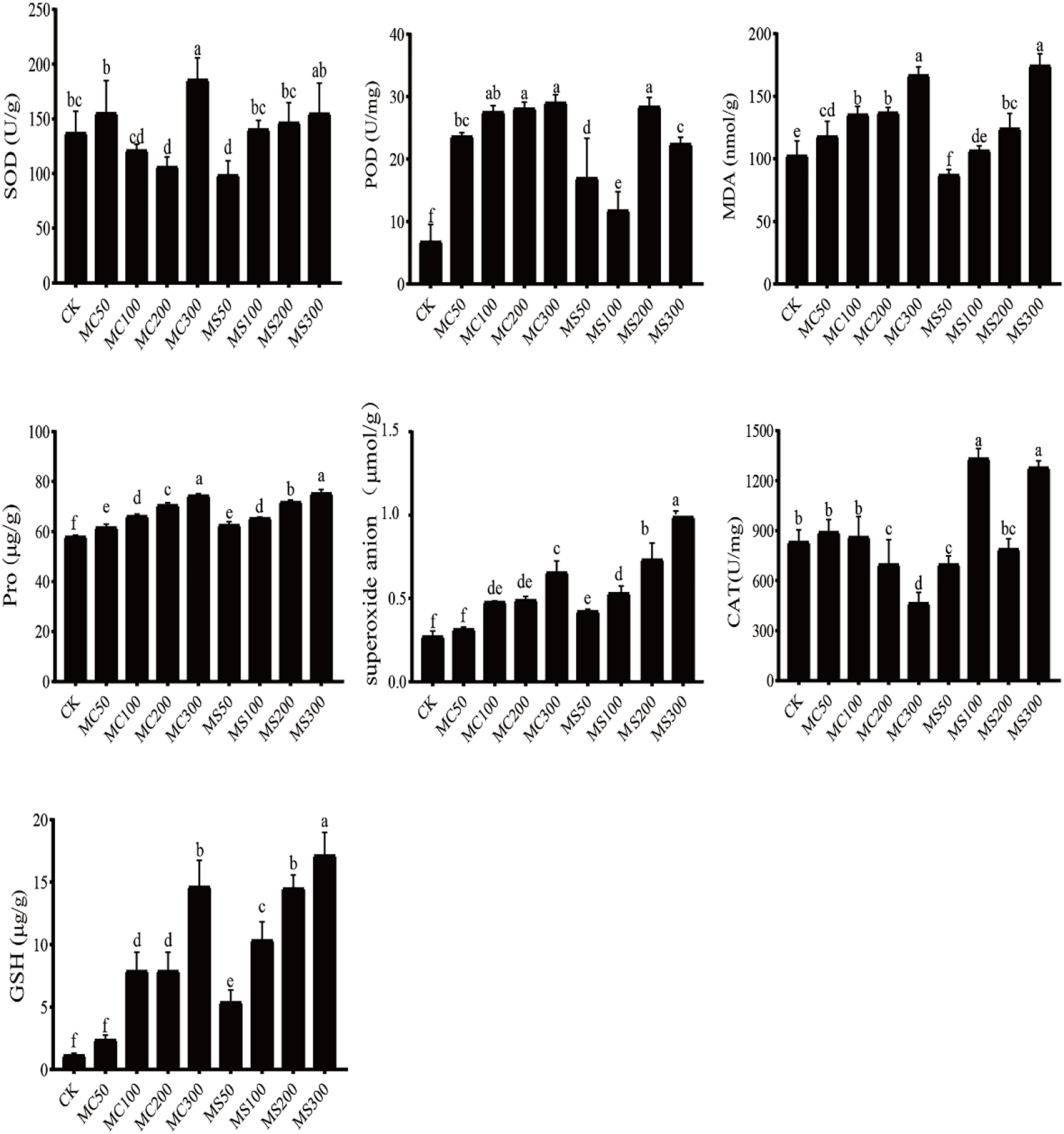
Physiological and biochemical indicators of *Y. henryi* with MgCl_2_ and MgSO_4_. CK, control; MC50/100/200/300, 50, 100, 200, and 300 mM MgCl₂ treatments; MS50/100/200/300, 50, 100, 200, and 300 mM MgSO₄ treatments. Different letters above the bars denote statistically significant differences among treatments at the 0.05 level. Two letters indicate that two treatments share the same letter

The effects of different MgCl₂ and MgSO₄ concentrations on physiological and biochemical parameters of *Y. henryi* leaf tissues were investigated (Fig. 10). Compared with CK, Superoxide Dismutase (SOD) activity decreased under MgCl₂ treatments from MC50 to MC200 but exceeded the control level at MC300. Under MgSO₄ treatments, SOD activity at MS50 was lower than CK, whereas it increased progressively from MS100 to MS300. Peroxidase (POD) activity generally increased with rising MgCl₂ concentrations (MC50-MC300). Similarly, POD activity in the MS50, MS200, and MS300 treatments was significantly higher than in the CK treatment. Malondialdehyde (MDA) content gradually increased under both MgCl₂ and MgSO₄ treatments, reaching maximum levels at MC300 and MS300. Free proline content was elevated under all Mg²⁺ treatments compared with CK, with the highest accumulation observed at both MC300 and MS300. The production rate of superoxide anions was higher under both MgCl₂ and MgSO₄ stress than in CK, with MgSO₄ treatments inducing faster production than MgCl₂. Catalase (CAT) activity showed significant differences relative to CK in all treatments except MC50. Under MgSO₄ stress, CAT activity declined as Mg²⁺ concentration increased, while the decomposition rate of hydrogen peroxide was maximal at 300 mM. Glutathione (GSH) content increased with enzyme activity and peaked at 300 mM. Overall, these results indicated that *Y. henryi* adjusted its physiological and biochemical responses to mitigate Mg²⁺-induced oxidative stress, thereby maintaining growth and development under high magnesium conditions.

### Subcellular localization of *YhNIPA* genes in *Y. henryi* protoplasts

The subcellular localization of the 17 *YhMGT* genes was predicted using the CELLO online tool (Table S9). The results showed that *YhMMgT* was localized in both the endoplasmic reticulum and mitochondria. *YhMRS2-1* was predicted to localize in chloroplasts and mitochondria, while *YhMRS2-2* was associated with chloroplasts and plastids. *YhMRS2-3* was mainly distributed in chloroplasts. Among the *YhMRS2* subfamily, *YhMRS2-4* was predicted to localize to mitochondria and vacuoles, whereas *YhMRS2-5/6/7* were predominantly mitochondrial proteins. For the *YhNIPA* subfamily, *YhNIPA1/2/3/4/8* were localized to the plasma membrane, while *YhMRS2-8*, and *YhNIPA5/6/7* were mainly distributed in chloroplasts.

Based on the RT-qPCR results, *YhNIPA3*/6/*8* were selected for subcellular localization analysis. Protoplasts of *Y. henryi* were successfully isolated using the method described [41]. The recombinant plasmids pCAMBIA1301-YhNIPA3-EGFP, pCAMBIA1301-YhNIPA6-EGFP, and pCAMBIA1301-YhNIPA8-EGFP were constructed, respectively. Two marker plasmids were also used: pCAMBIA1300-AtPIP2A-mCherry for plasma membrane localization and pCAMBIA1300-AtRBSC-mCherry for chloroplast localization. The primers used to construct the vector described above are shown in Table S10.

**Fig. 11:**
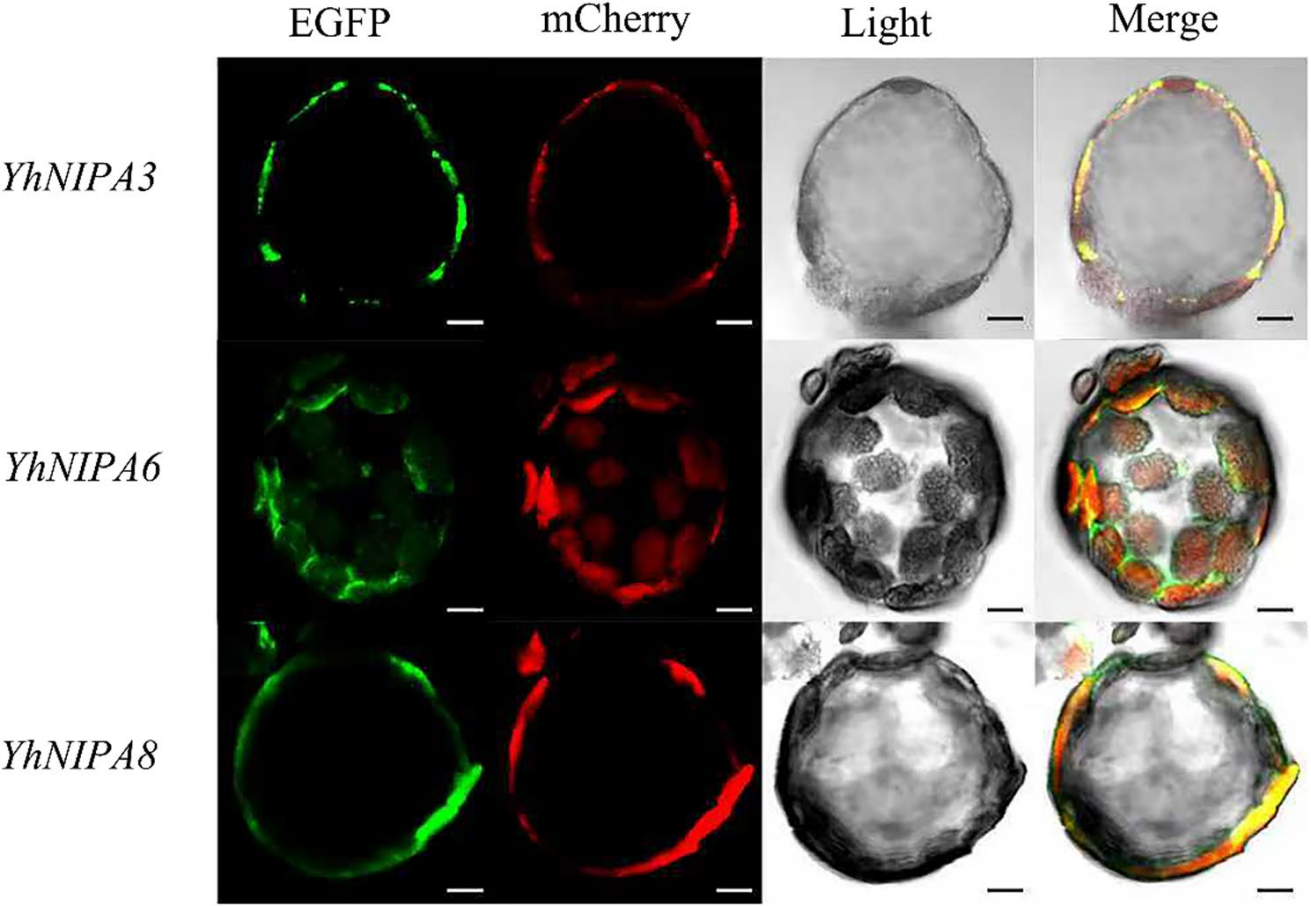
Subcellular localization of protoplasts of *Y. henryi*. YhNIPA3-EGFP, YhNIPA8-EGFP, and AtPIP2A-mCherry fusion proteins were co-expressed, and YhNIPA6-EGFP and AtRBSC-mCherry fusion protein were co-expressed in the protoplast of *Y.henryi*. The left-to-right bar charts display signals of Enhanced Green Fluorescent Protein (EGFP), monomeric Cherry Fluorescent Protein (mCherry), and combined images of light, EGFP, and mCherry. Bar = 5 μm. Vector construction assembly in Fig. S6

In protoplasts of *Y. henryi*, pCAMBIA1301-YhNIPA3-EGFP and pCAMBIA1301-YhNIPA8-EGFP were co-transfected with pCAMBIA1300-AtPIP2A-mCherry, while pCAMBIA1301-YhNIPA6-EGFP was co-transfected with pCAMBIA1300-AtRBSC-mCherry. The samples were examined under confocal laser scanning microscopy to detect fluorescence signals. The green fluorescence of YhNIPA3-EGFP and YhNIPA8-EGFP overlapped with the red fluorescence of AtPIP2A-mCherry, producing a yellow signal on the plasma membrane, indicating their membrane localization. In contrast, YhNIPA6-EGFP signals showed strong co-localization with AtRBSC-mCherry, confirming its localization in chloroplasts (Fig. 11). Overall, these results demonstrated that *YhNIPA3* and *YhNIPA8* were localized on plasma membrane, whereas *YhNIPA6* was localized on chloroplasts.

## Discussions

Plants acquire Mg from the soil and distribute it among organs, cells, and subcellular compartments primarily through Mg²⁺ transporters, which are essential for maintaining Mg²⁺ homeostasis [3, 42]. In *Arabidopsis*, different *MGT* family members display tissue-specific expression and subcellular localization, reflecting functional specialization in Mg²⁺ uptake, translocation, and compartmentalization [8, 43–45]. In addition, Magnesium Release (MGR) proteins function primarily in Mg²⁺ efflux and intracellular redistribution, further contributing to Mg²⁺ homeostasis [3, 46, 47]. Similarly, the 17 *YhMGT* genes identified in *Y. henryi* were classified into three subfamilies (*MRS2*, *NIPA*, and *MMgT*), consistent with the conserved organization of *MGT* families in higher plants. The presence of the GMN motif in YhMRS2 proteins suggests functional conservation [48], whereas differences in transmembrane domains among YhMRS2, YhNIPA, and YhMMgT proteins indicate potential functional diversification. Phylogenetic analysis revealed closer clustering with dicots, particularly *Arabidopsis*, highlighting evolutionary conservation. Notably, *YhMRS2-1* grouped with *AtMRS2-7*, a gene critical under low-Mg conditions [49], suggesting a role in Mg acquisition under limiting environments. NIPA proteins in plants remain less characterized, but the complex exon-intron structures observed in *YhNIPA* genes may reflect conserved regulatory strategies and functional plasticity [18, 50].

Expression profiling under MgCl₂ and MgSO₄ stress revealed clear anion-dependent transcriptional responses of *YhMGT* genes. Most *YhMGT* genes were strongly induced under moderate Mg concentrations (200 mM), suggesting activation of Mg²⁺-specific transport and redistribution mechanisms. In contrast, excessive Mg treatment (300 mM) resulted in attenuated or plateaued *YhMGT* expression, indicating that Mg²⁺ homeostatic regulation may approach its upper regulatory limit under extreme conditions. Similar Mg stress differentiation has been reported in *Arabidopsis* and *Malus domestica* [8, 51]. Notably, GO enrichment analysis showed that osmotic stress-related pathways were specifically enriched under MS300 but not MC300 treatment (Table 4; Fig. S4J), suggesting that general osmotic stress responses are more closely associated with high sulfate supply rather than chloride-associated Mg²⁺ stress. This distinction indicates that transcriptional responses under extreme Mg²⁺ conditions may arise from different physiological drivers depending on the accompanying anion. While MS300 likely triggers a combination of Mg²⁺ toxicity and osmotic stress, MC300 responses appear to be dominated by Mg²⁺-specific or Cl⁻-related regulatory mechanisms. Collectively, these findings suggest that *Y. henryi* has evolved a finely tuned Mg²⁺ regulatory network that discriminates between Mg²⁺-specific regulation and anion-associated stress signals, thereby enhancing tolerance to diverse Karst Mg environments.

While cation transporters have been extensively studied for their roles in maintaining ionic homeostasis, the contribution of anion transport systems to this process remains relatively underexplored [52]. Under salt stress, plants maintain cellular ionic and osmotic balance through coordinated regulation of both cation and anion fluxes [53]. Chloride channels (CLCs), primarily localized to intracellular membranes, contribute to Cl⁻ sequestration and redistribution, whereas sulfate uptake and intracellular transport are mediated by SULTR family members, linking sulfate availability with sulfur assimilation and chloroplast function [54–56]. In this study, MgCl₂ and MgSO₄ treatments elicited distinct transcriptional responses of *YhMGT* genes, with MgCl₂ inducing maximal expression at 200 mM, while MgSO₄ triggered peak expression at lower concentrations. Consistently, RNA-seq analysis showed that most *YhCLC* genes were upregulated under high Mg²⁺ treatment, whereas the majority of *YhSULTR* genes exhibited a general downregulation trend (Fig. S7). These contrasting patterns suggest that chloride- and sulfate-associated Mg stress may differentially influence *YhMGT* regulation through distinct anion transport pathways. Sulfate, as an essential macronutrient, participates in redox metabolism and biosynthetic processes, which may indirectly modulate *YhMGT* transcription under MgSO₄ treatment. In contrast, excessive chloride accumulation induces oxidative stress and activate abscisic acid (ABA)-dependent signaling pathways. The presence of ABA-responsive and MYB-binding cis-elements in *YhMGT* promoters suggests that Cl⁻-induced signaling may transcriptionally enhance *YhMGT* expression to facilitate Mg²⁺ transport and alleviate ionic toxicity. Moreover, the stronger ionic association between Mg²⁺ and SO₄²⁻ compared with Cl⁻ may reduce the accumulation of free Mg²⁺ in cells, contributing to differences in Mg sensing and transporter regulation. Collectively, these findings indicate that Cl⁻ and SO₄²⁻ transport systems may act coordinately with *YhMGT*s to fine-tune Mg²⁺ homeostasis in *Y. henryi*.

Subcellular localization analysis further supports functional specialization among *YhMGT* family members. Previous studies have shown that MGT proteins are localized to diverse cellular compartments across species, including the plasma membrane, endoplasmic reticulum, chloroplast, and nucleus, reflecting their distinct physiological roles [14, 42, 51]. In *Y. henryi*, *YhNIPA3* and *YhNIPA8* were predominantly localized to the plasma membrane, suggesting roles in Mg²⁺ uptake from the external environment, whereas *YhNIPA6* was localized to the chloroplast, indicating involvement in intracellular Mg²⁺ redistribution. Such compartment-specific localization implies a coordinated Mg²⁺ transport system operating across multiple cellular membranes. In particular, chloroplast-localized *YhNIPA6* may contribute to maintaining chloroplastic Mg²⁺ supply, thereby supporting photosynthetic stability under Mg stress.

Plants inhabiting karst ecosystems often evolve specialized physiological and molecular mechanisms to cope with high and fluctuating concentrations of divalent cations, particularly Ca²⁺ and Mg²⁺ [57, 58]. Previous studies have demonstrated that *Y. henryi* exhibits notable tolerance to elevated calcium levels [24], and the present results indicate a similar tolerance to high Mg²⁺ conditions. RNA-seq and RT-qPCR analysis revealed that *YhMMgT*, *YhMRS2-2/6/8*, and *YhNIPA3/5/8*, maintained relatively high expression levels under high Mg treatment. In the context of karst habitats, *YhMGT* genes exhibiting sustained induction under Mg stress together with strategic subcellular localization—particularly plasma membrane–localized *YhNIPA3* and *YhNIPA8*—are likely key components of adaptive Mg²⁺ regulation. In addition, other Mg-responsive members, including *YhMMgT* and *YhMRS2-2/6/8*, which showed consistently elevated expression under high Mg²⁺ conditions, may act cooperatively to support intracellular Mg²⁺ transport and compartmental homeostasis. The differential responsiveness of these genes to MgCl₂ and MgSO₄ further suggests that distinct anion environments may selectively recruit specific *YhMGT* members within this regulatory network. Such a multi-gene regulatory framework likely provides functional redundancy and flexibility, enabling *Y. henryi* to fine-tune Mg²⁺ uptake, redistribution, and organellar supply in Mg-rich and ionically complex karst environments.

In conclusion, this study provides a comprehensive characterization of the *YhMGT* gene family and reveals anion-dependent transcriptional regulation in response to Mg²⁺ stress, offering insights into magnesium homeostasis in *Y. henryi*. Future work employing transgenic approaches, CRISPR-mediated editing, and protein-protein interaction analyses will be critical to elucidate the underlying molecular mechanisms and regulatory networks that enable adaptation to Mg-rich karst environments.

## Conclusions

*Y. henryi*, a karst-endemic species, exhibits unique adaptations that enable tolerance to high magnesium levels in its native soils. This study provides a comprehensive characterization of the *YhMGT* gene family and reveals their roles in magnesium homeostasis. The main conclusions are:

1. Seventeen *YhMGT* genes were identified and classified into three subfamilies (*MRS2*, *NIPA*, *MMgT* ), each exhibiting conserved motifs and domain structures, reflecting both evolutionary conservation and potential functional diversification.
2. *YhMGT* genes are unevenly distributed across six chromosomes, with some arranged in tandem clusters. Promoter analysis revealed multiple cis-elements associated with stress responses and hormonal regulation, suggesting complex transcriptional control.
3. RNA-seq and RT-qPCR analyses demonstrated that *YhMGT* gene expression is differentially regulated by Mg²⁺ supplied as MgCl₂ versus MgSO₄, highlighting anion-dependent modulation of magnesium transporters in *Y. henryi*.
4. Subcellular localization revealed that *YhNIPA6* is localized in the chloroplast, while *YhNIPA3* and *YhNIPA8* are localized in the plasma membrane.
5. The coordinated expression of *YhMGT* genes enables selective Mg²⁺ transport, mitigates potential ionic toxicity, and supports photosynthesis and energy metabolism, providing a molecular basis for adaptation to high-Mg karst soils.

Overall, this study advances our understanding of magnesium transporter regulation under distinct anionic conditions and offers insights into the molecular mechanisms underlying karst plant adaptation.

## Supplementary Information

Supplementary Material 1.

## Data Availability

The datasets generated during the current study are accessible from the corresponding author upon reasonable request. Publicly available resources include the whole-genome sequencing data (CNCB, accession CRA019022, https://www.cncb.ac.cn/search?dbId=andq=CRA019022) and the RNA-seq data (GeneBank, accession PRJNA1172955, https://www.ncbi.nlm.nih.gov/sra/?term=PRJNA1172955).

## Abbreviations

ABA: Abscisic Acid
CLCs: Chloride channels
CNCB: China National Center for Bioinformation database
CDD: NCBI Conserved Domain Database
DUF803: Domain of Unknown Function 803
DEGs: Differentially Expressed Genes
EGFP: Enhanced Green Fluorescent Protein
GO: Gene Ontology
GMN: Gly-Met-Asn motif
KEGG: Kyoto Encyclopedia of Genes and Genomes
ML: Maximum Likelihood
MGT: Magnesium transporter
MGR: Magnesium Release
MRS2: Magnesium transporter MRS2
MMgTs: Membrane Mg²⁺ transporters
MgCl₂: Magnesium chloride
MgSO₄: Magnesium sulfate
mCherry: Monomeric Cherry Fluorescent Protein
NIPA: Non-imprinted in Prader–Willi/Angelman syndrome family
RNA-Seq: RNA sequencing
ROS: Reactive oxygen species
SULTR: SULTR transporter family
