# Supplementary material for: Genome-wide identification and functional characterization of the Magnesium Transporter (MGT) gene family and its expression patterns to different anionic magnesium stresses in Yinshania henryi
Source: BMC Genomics. 2026 Mar 2;27:356. doi: 10.1186/s12864-026-12704-z (PMC13059214; doi:10.1186/s12864-026-12704-z)
Supplement: Supplementary file 1 — Supplementary Material 1. [file 12864_2026_12704_MOESM1_ESM.zip › Supplementary Files/Figure S1.docx]

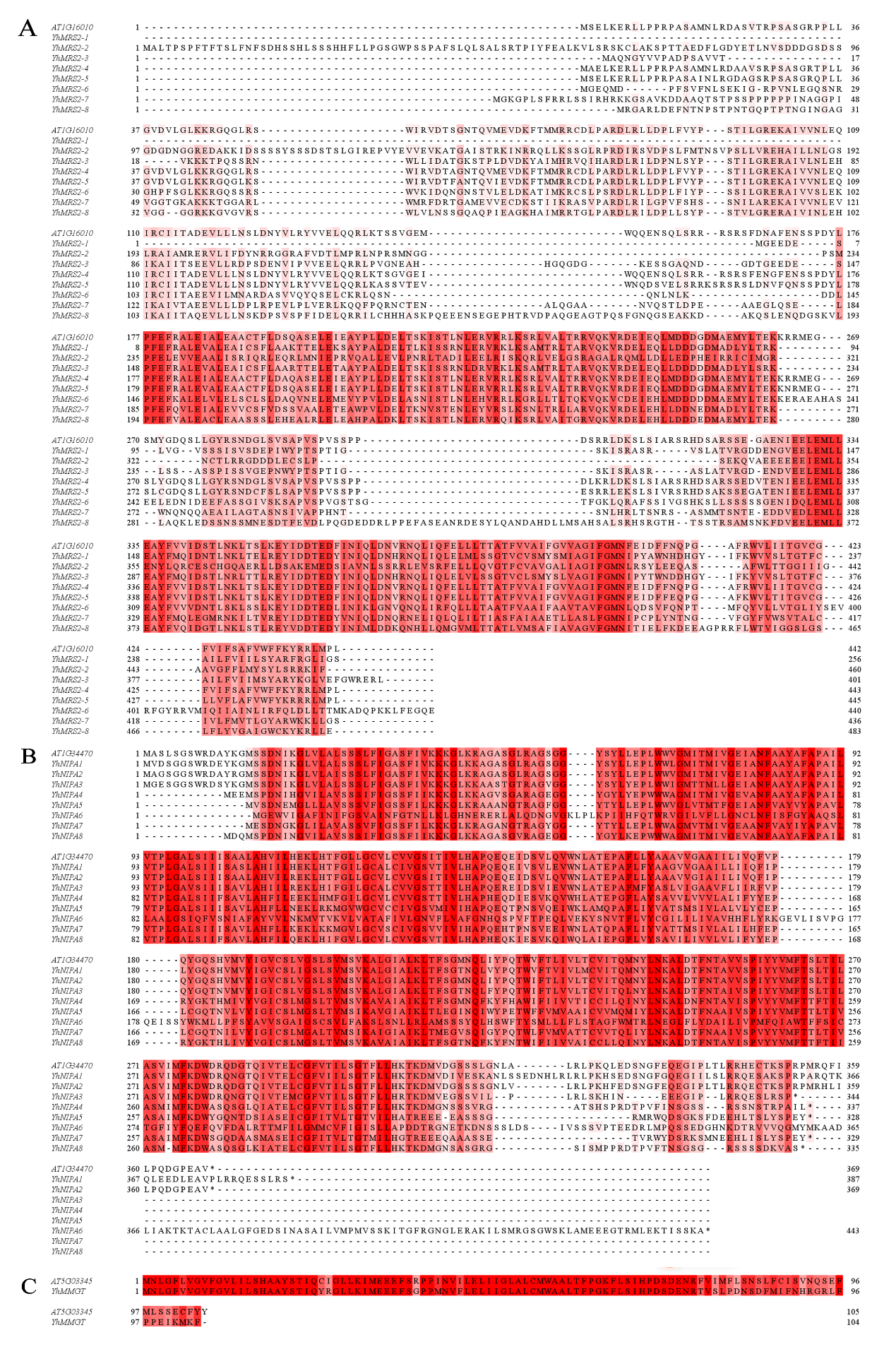


**Figure S1.** Alignment of 17 YhMGT proteins and selected AtMGT proteins. (A) YhMRS2 proteis. (B) YhNIPA proteins. (C)YhMMgT protein.
