# Supplementary material for: Genome-wide identification and functional characterization of the Magnesium Transporter (MGT) gene family and its expression patterns to different anionic magnesium stresses in Yinshania henryi
Source: BMC Genomics. 2026 Mar 2;27:356. doi: 10.1186/s12864-026-12704-z (PMC13059214; doi:10.1186/s12864-026-12704-z)
Supplement: Supplementary file 1 — Supplementary Material 1. [file 12864_2026_12704_MOESM1_ESM.zip › Supplementary Files/Figure S4.docx]

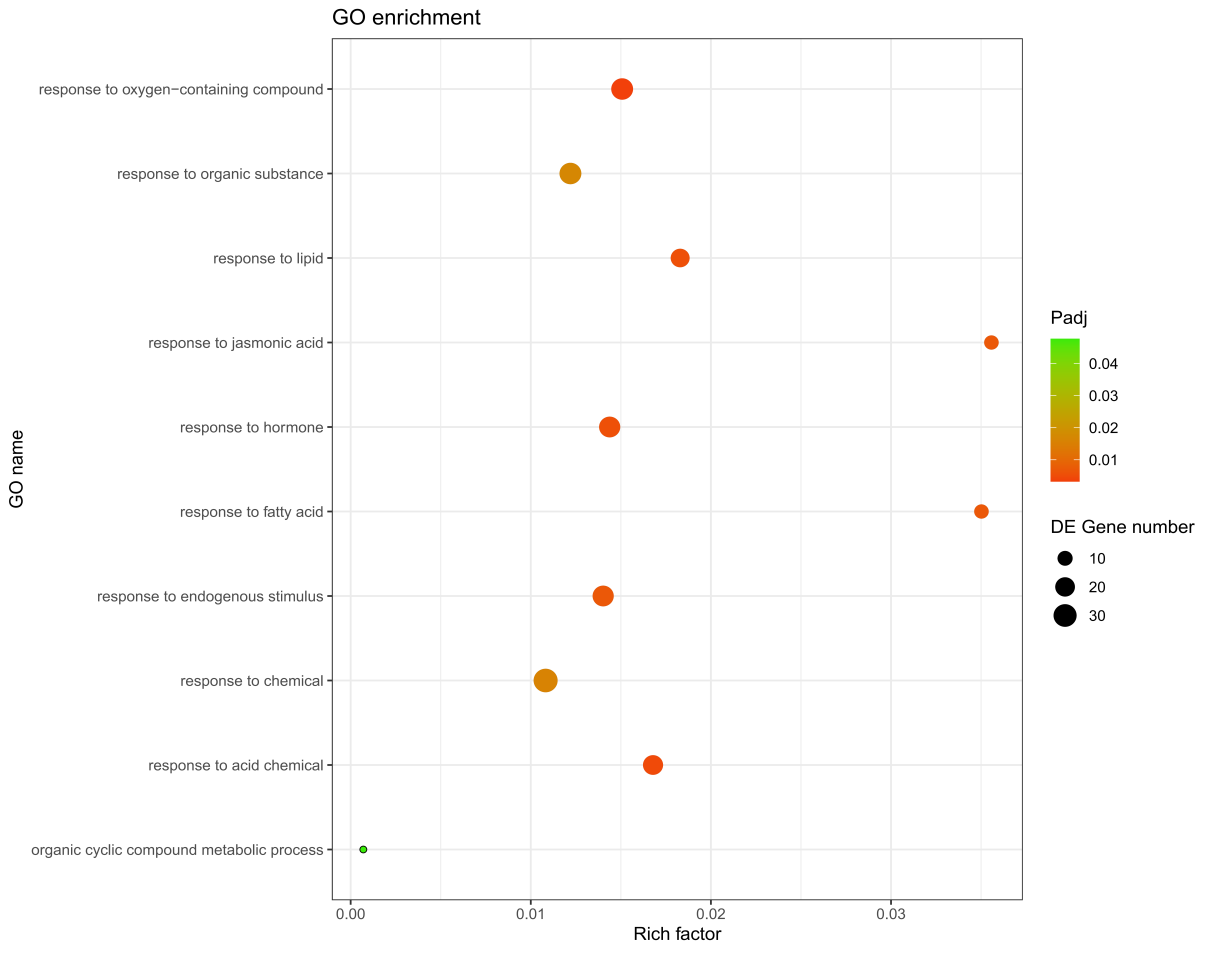


A

Biological Process of CK vs. MC100


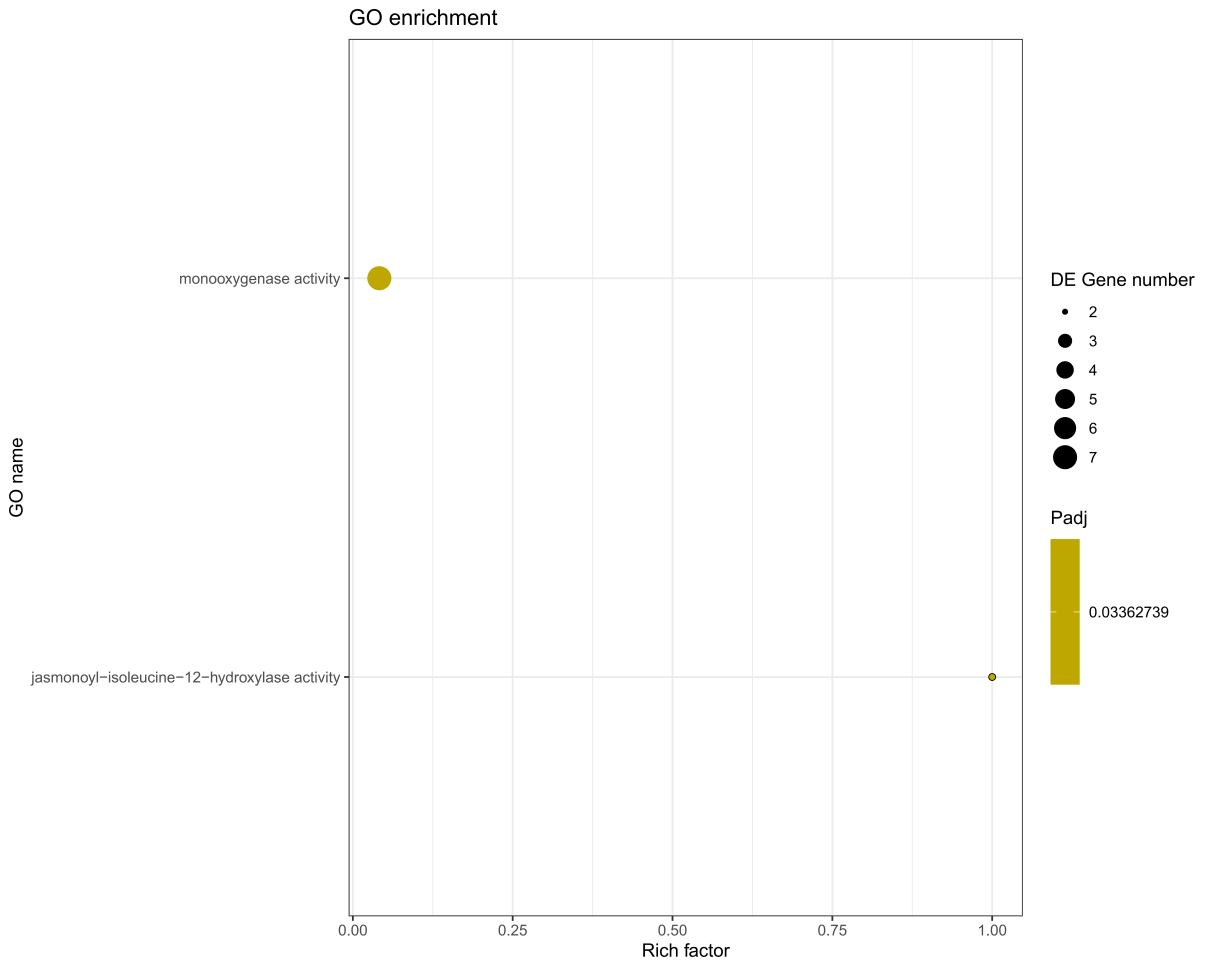


B

Molecular Function of CK vs. MC100


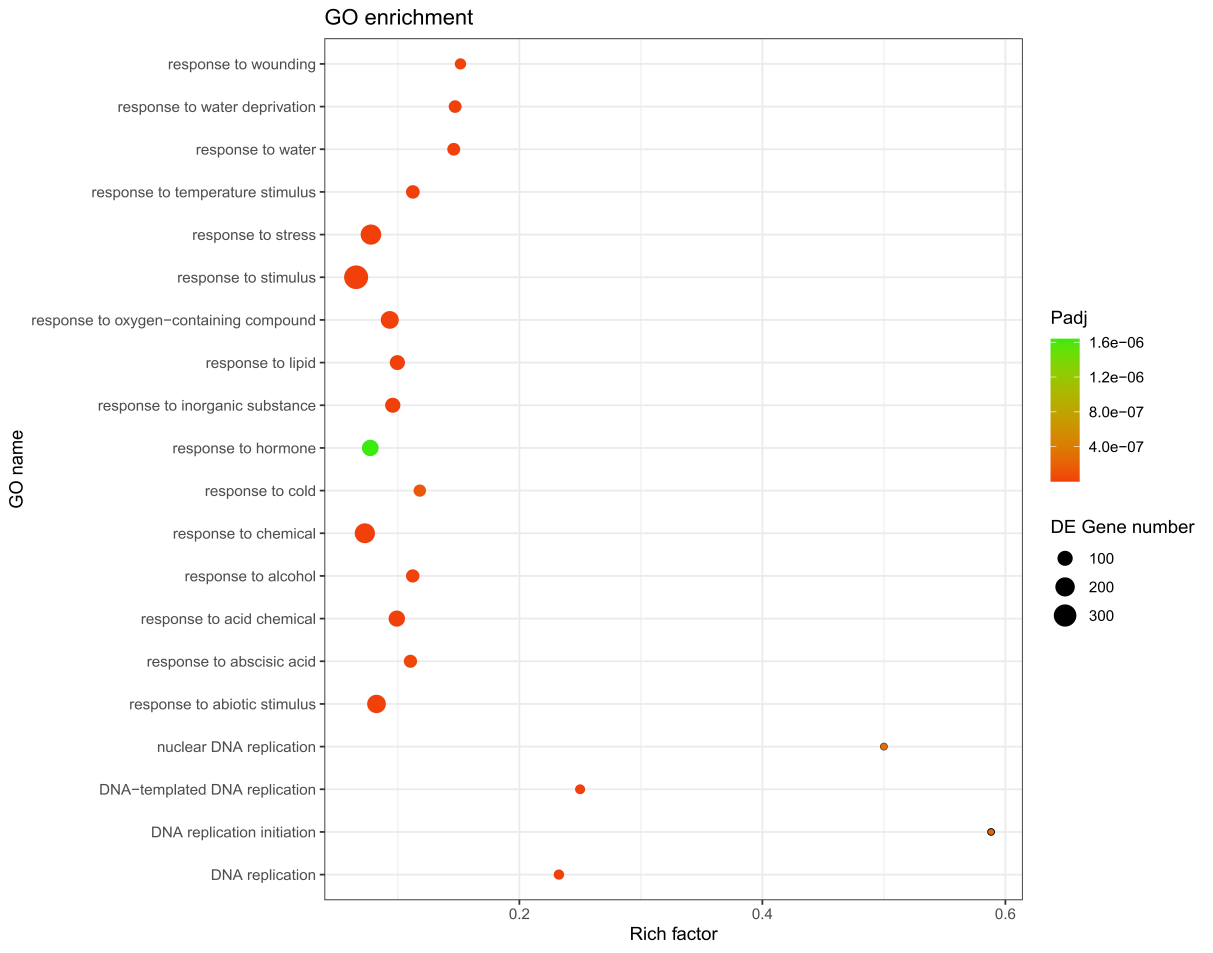


C

Biological Process of CK vs. MC200


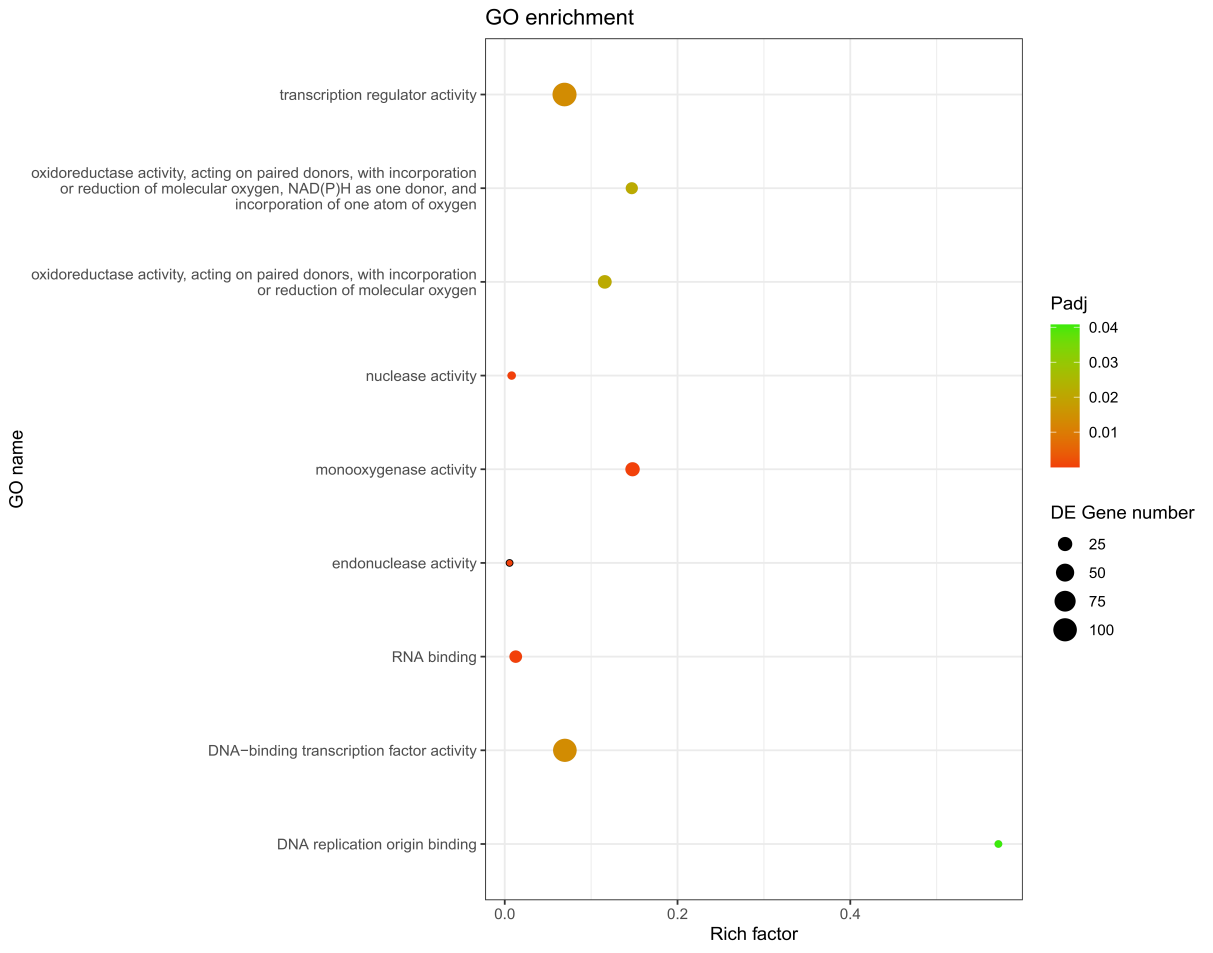


D

Molecular Function of CK vs. MC200


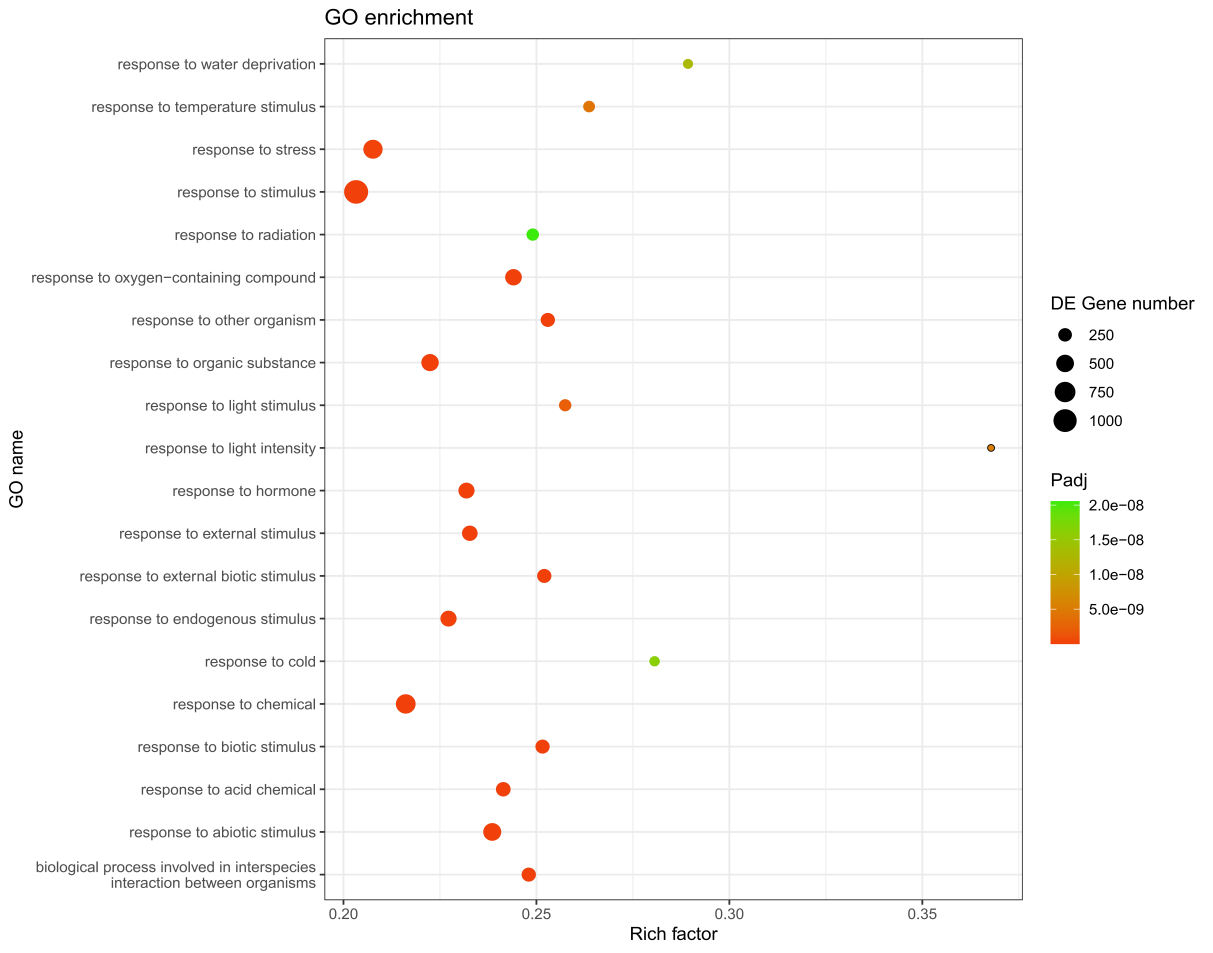


E

Biological Process of CK vs. MC300


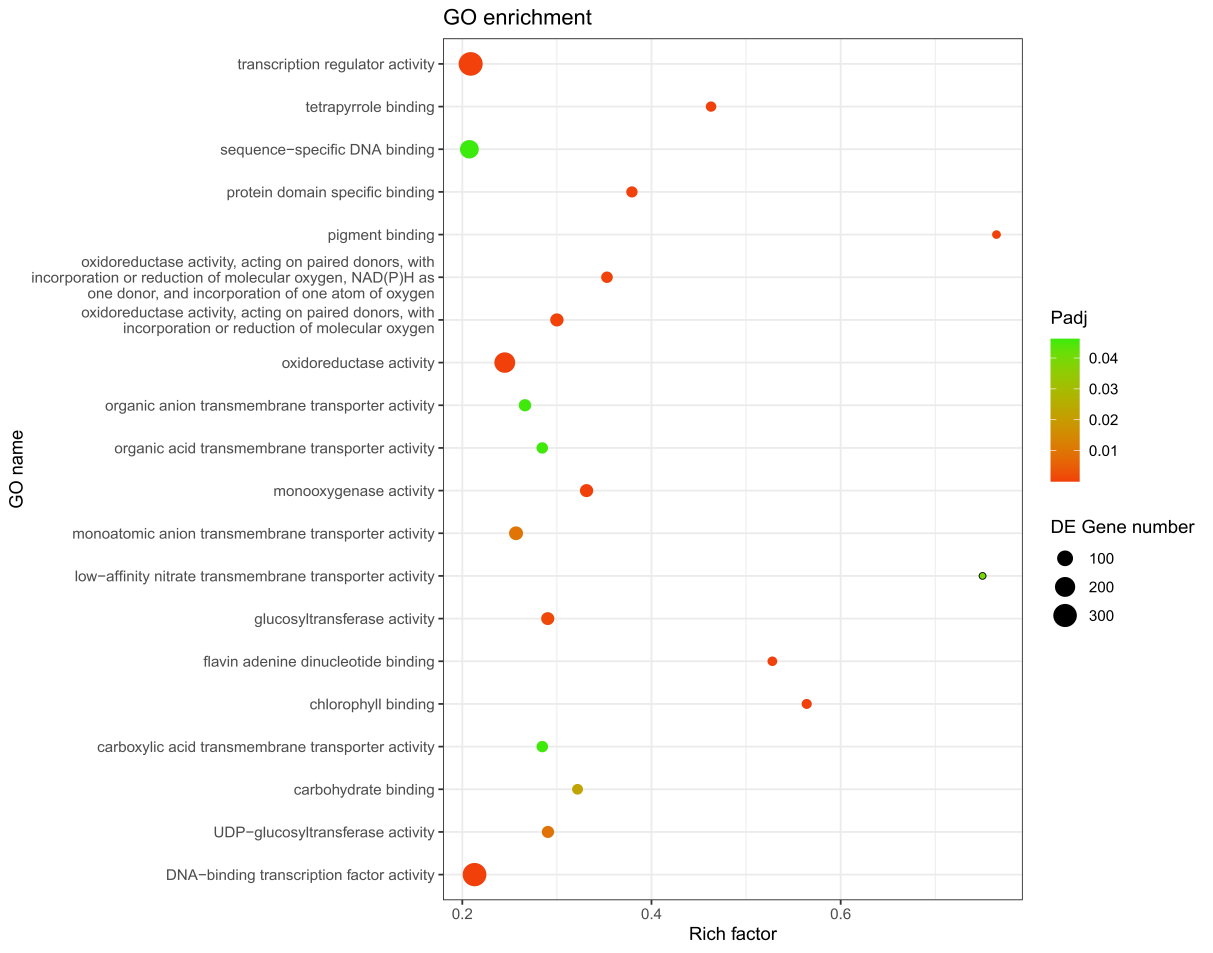


F

Molecular Function of CK vs. MC300


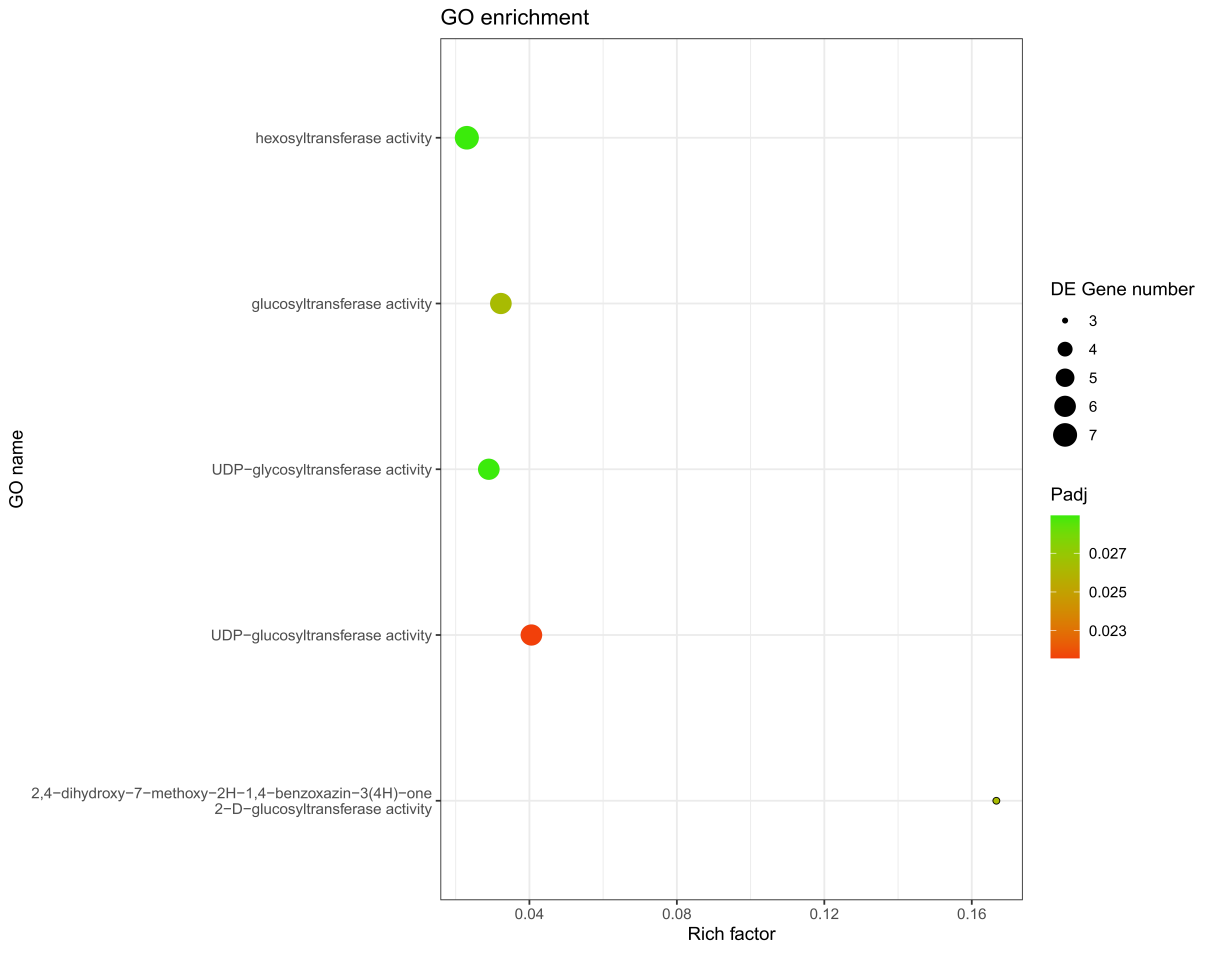


G

Molecular Function of CK vs. MS100


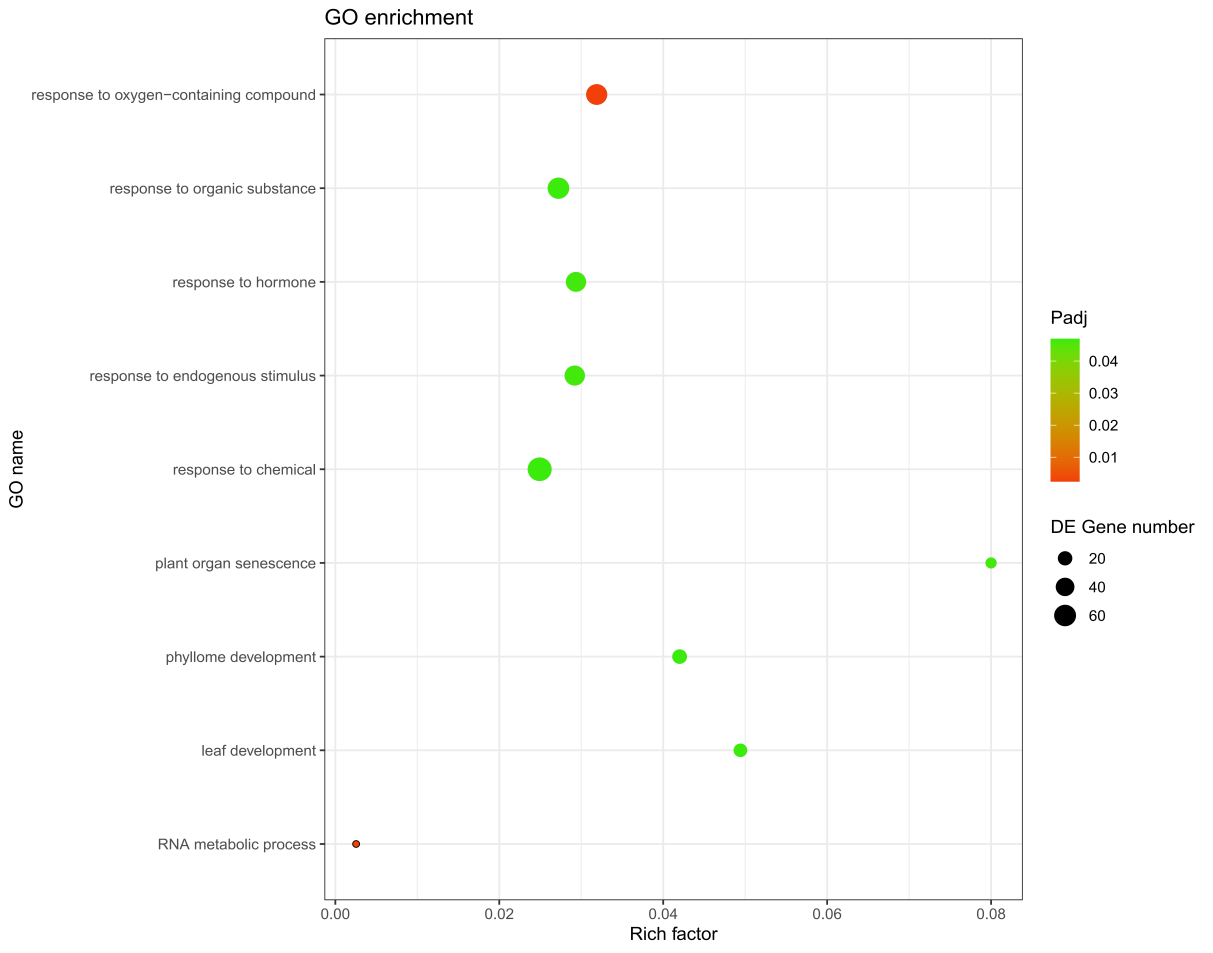


H

Biological Process of CK vs. MS200


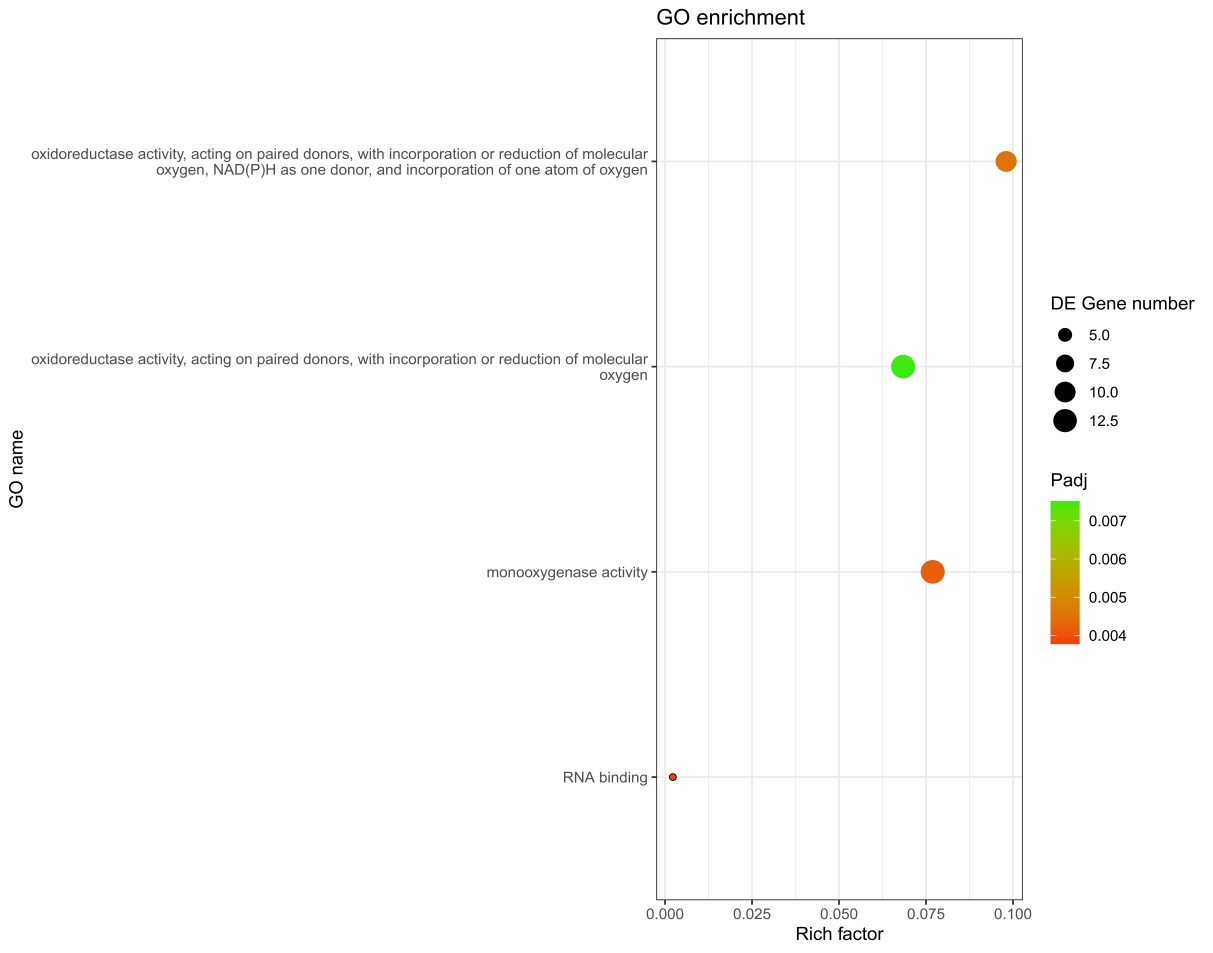


I

Molecular Function of CK vs. MS200


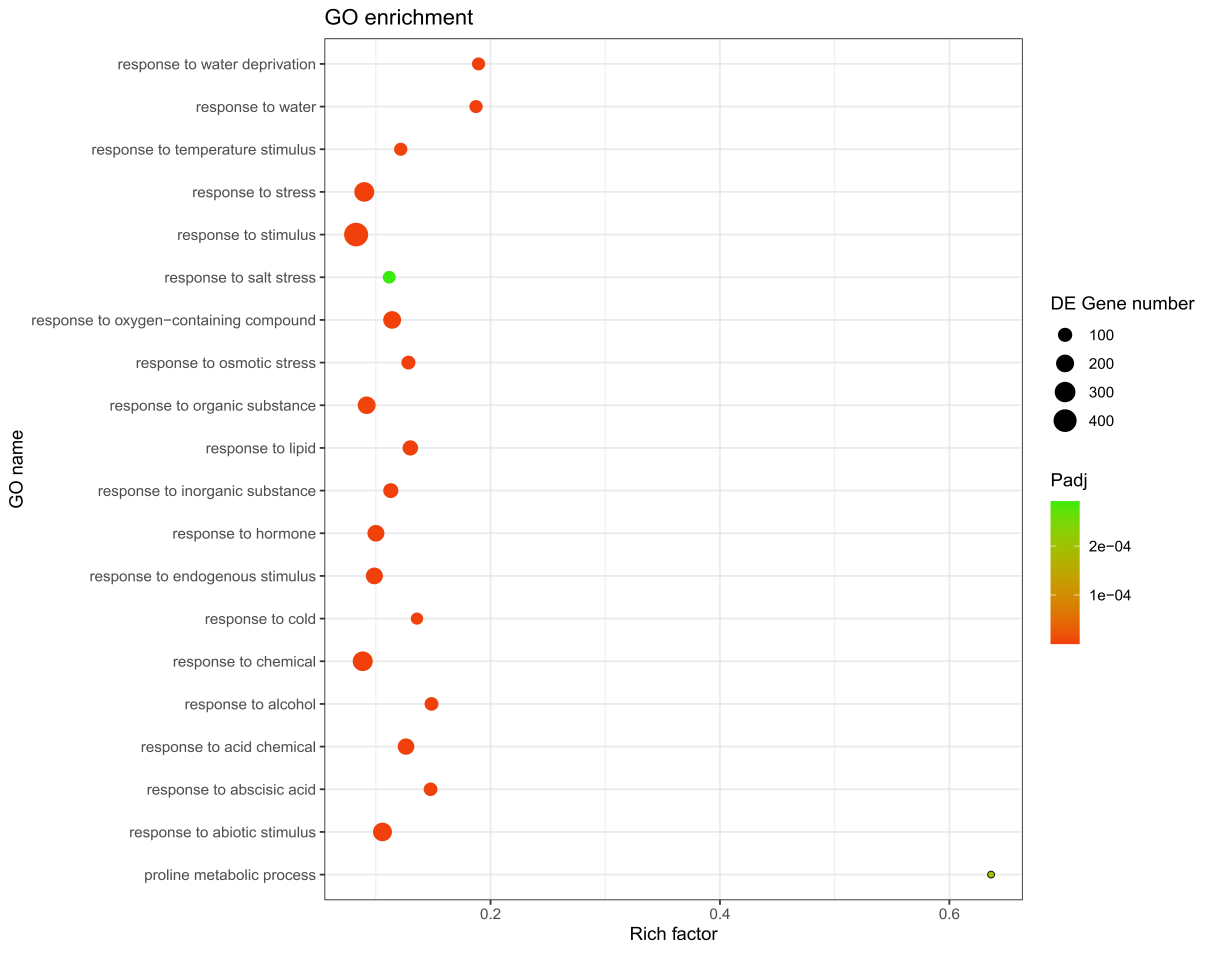


J

Biological Process of CK vs. MS300





K

Molecular Function of CK vs. MS300

**Figure S4.** GO terms significantly enriched among the DEGs identified across different treatment groups. (A) Biological Process of CK vs. MC100; (B) Molecular Function of CK vs. MC100; (C) Biological Process of CK vs. MC200; (D) Molecular Function of CK vs. MC200; (E) Biological Process of CK vs. MC300; (F) Molecular Function of CK vs. MC300; (G) Molecular Function of CK vs. MS100; (H) Biological Process of CK vs. MS200; (I) Molecular Function of CK vs. MS200; (J) Biological Process of CK vs. MS300; (K) Molecular Function of CK vs. MS300.
