# Supplementary material for: Genome-wide identification and functional characterization of the Magnesium Transporter (MGT) gene family and its expression patterns to different anionic magnesium stresses in Yinshania henryi
Source: BMC Genomics. 2026 Mar 2;27:356. doi: 10.1186/s12864-026-12704-z (PMC13059214; doi:10.1186/s12864-026-12704-z)
Supplement: Supplementary file 1 — Supplementary Material 1. [file 12864_2026_12704_MOESM1_ESM.zip › Supplementary Files/Figure S5.docx]

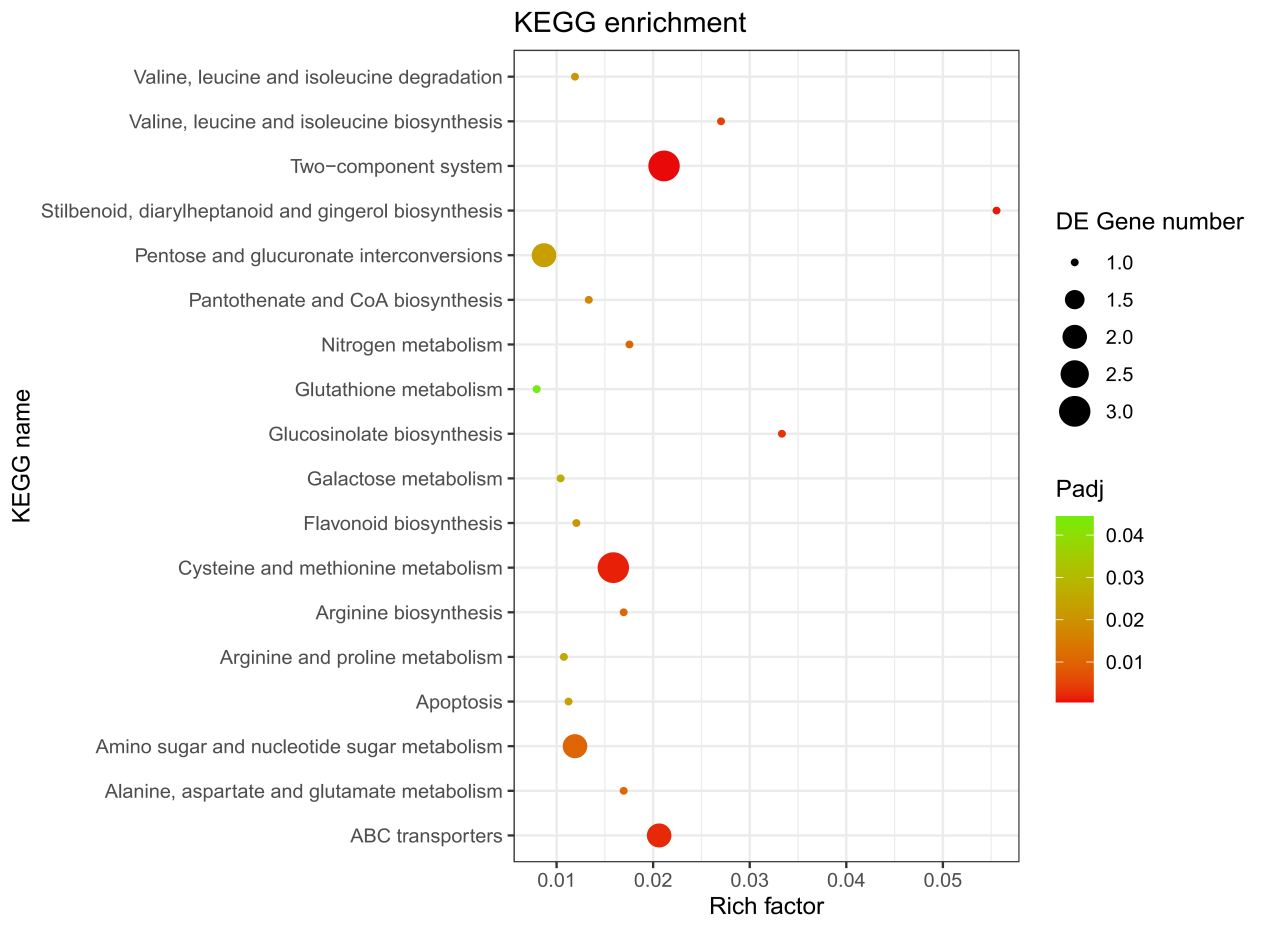


A

CK vs MC50

B


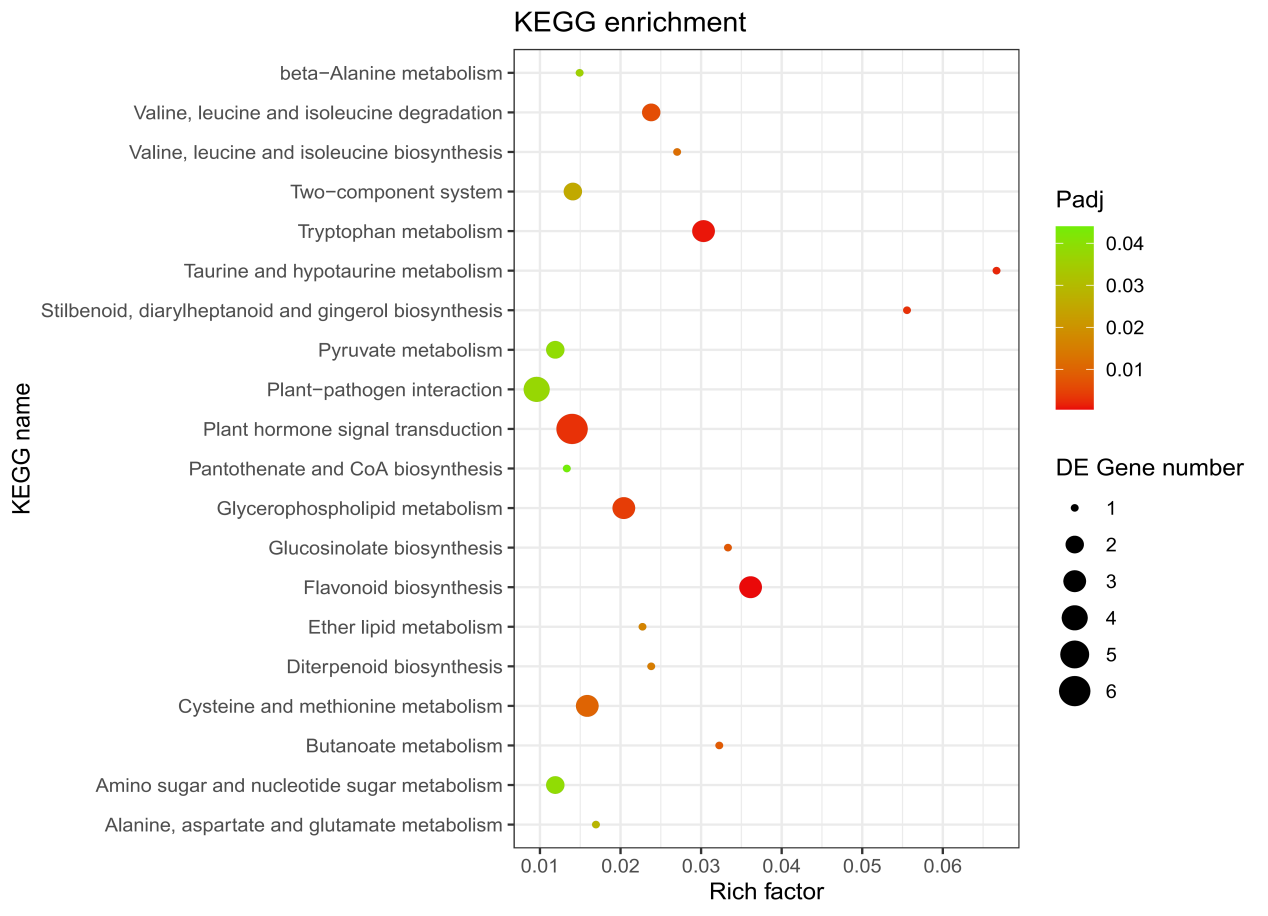


CK vs MC100


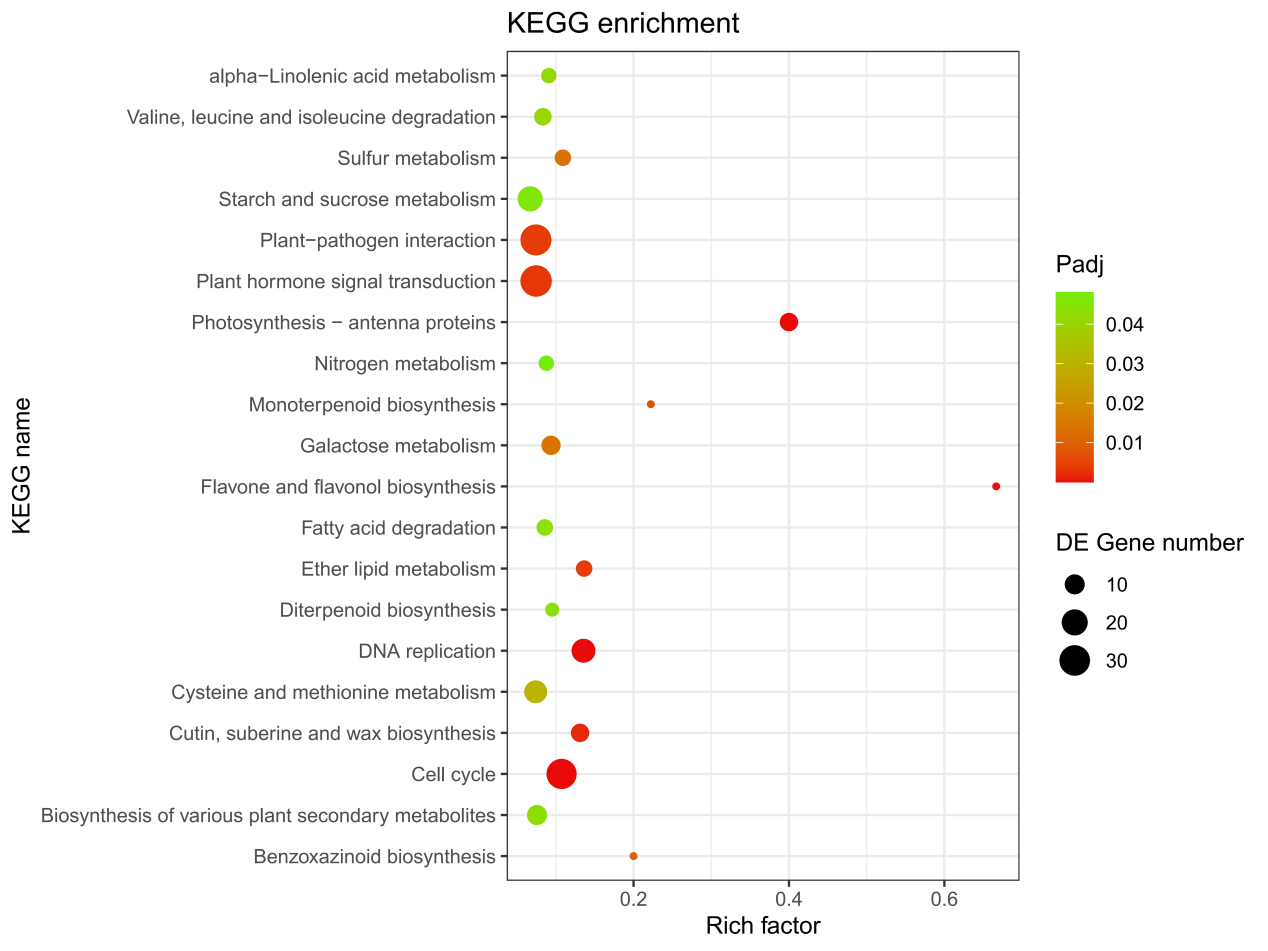


C

CK vs MC200


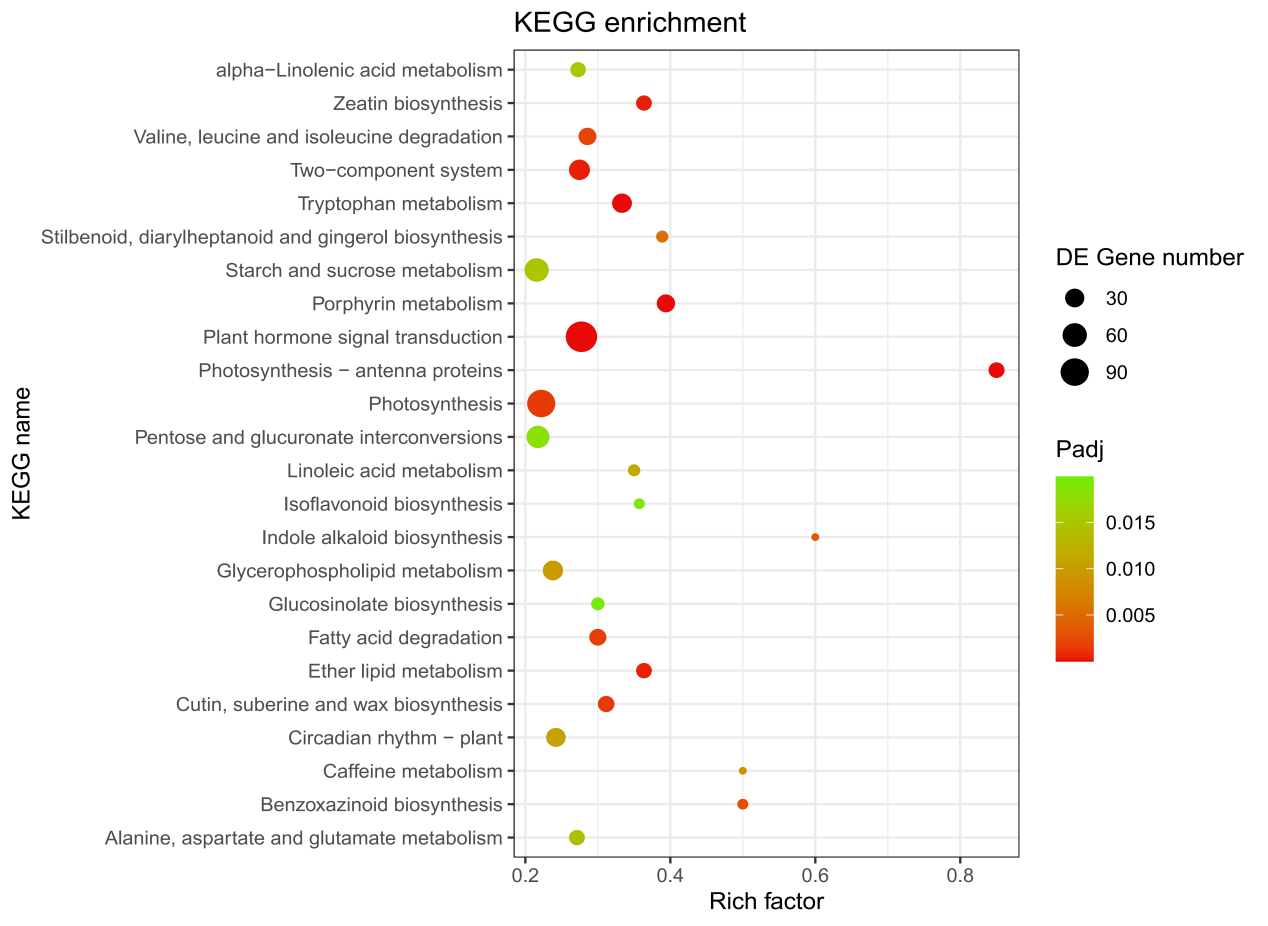


D

CK vs MC300


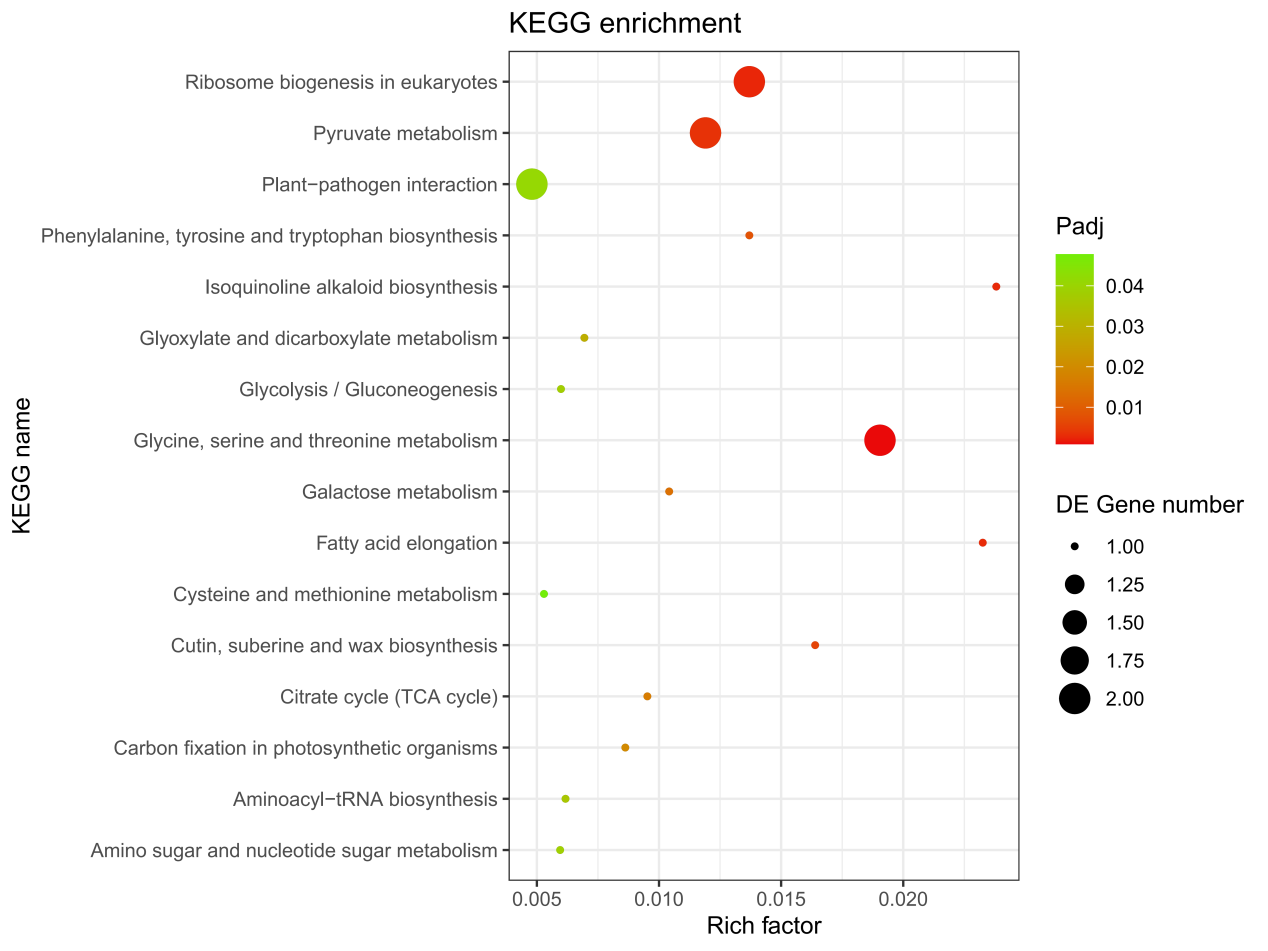


E

CK vs MS50


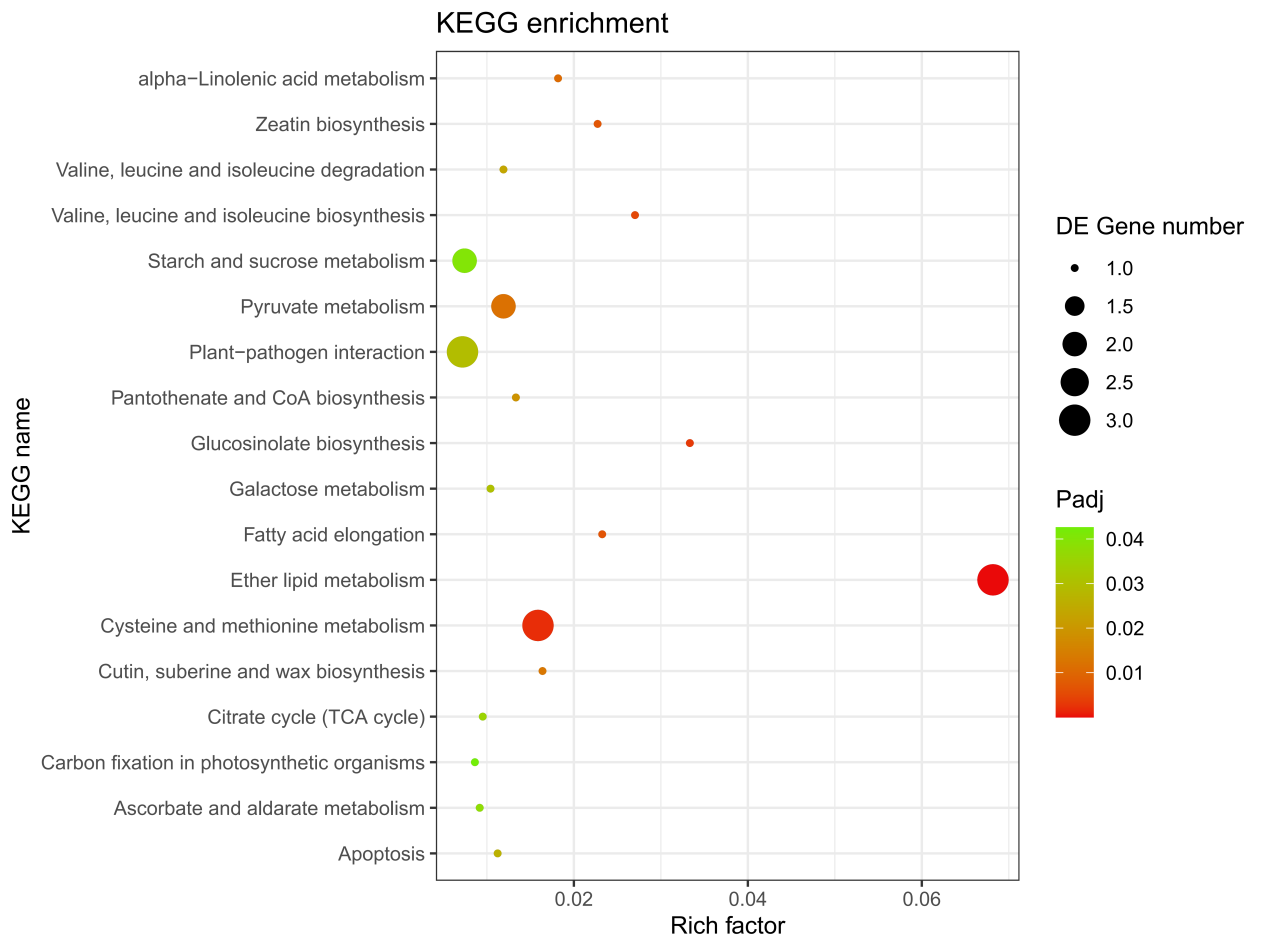


F

CK vs MS100


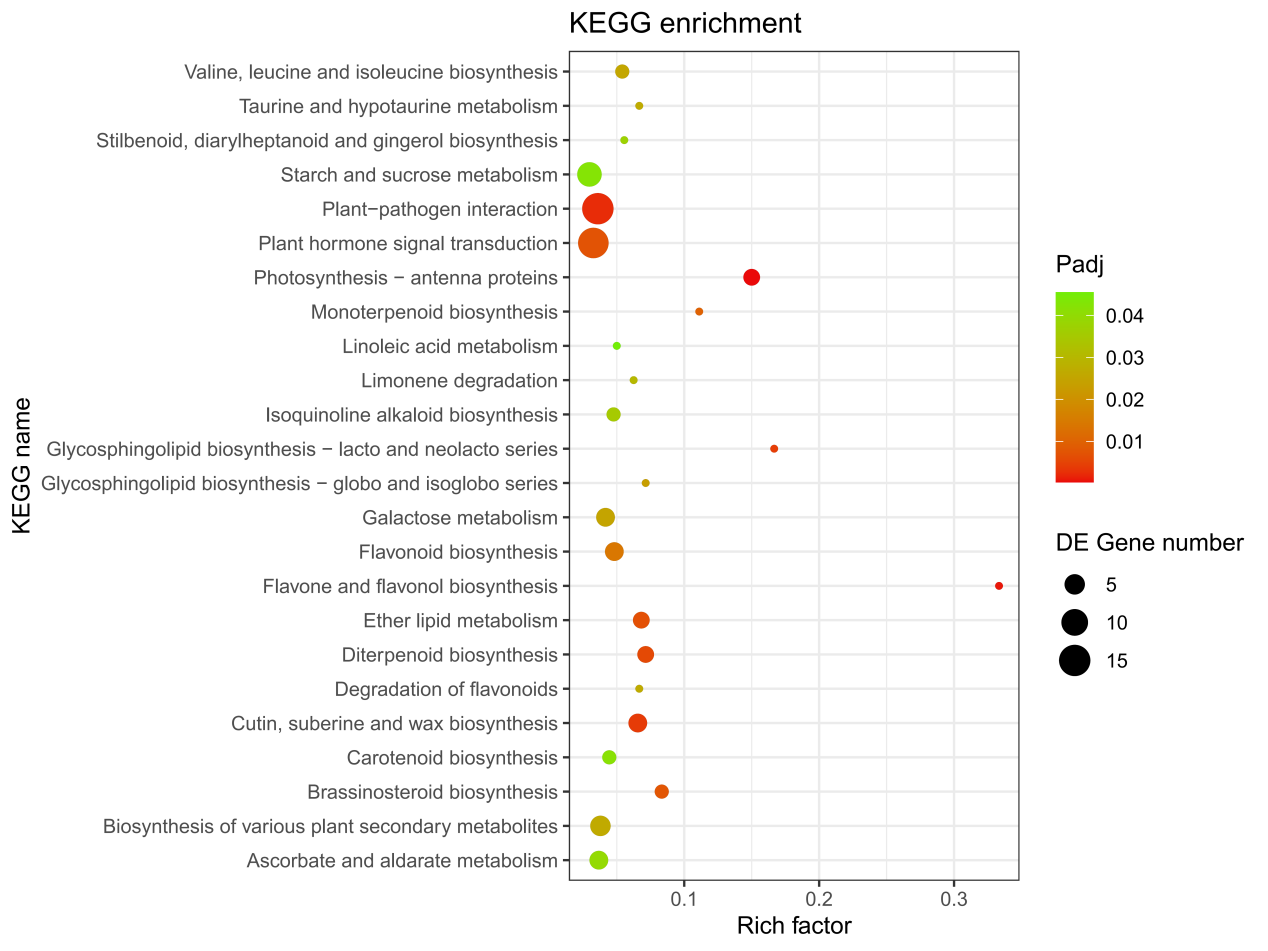


G

CK vs MS200


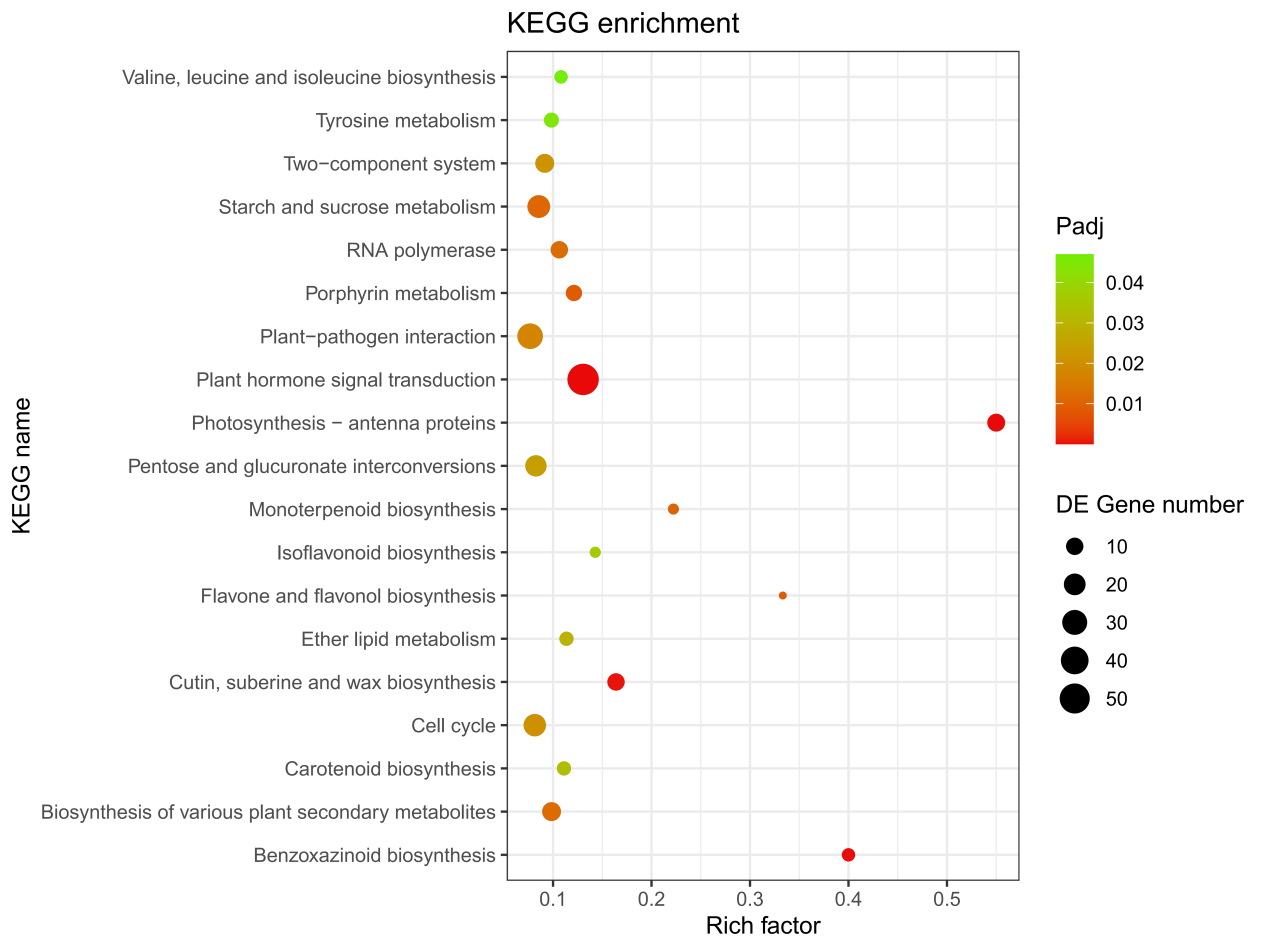


H

CK vs MS300

**Fig. S5.** KEGG pathways significantly enriched among the DEGs identified across different treatment groups. (A) CK vs. MC50; (B) CK vs. MC100; (C) CK vs. MC200; (D) CK vs. MC300; (E) CK vs. MS50; (F) CK vs. MS100; (G) CK vs. MS200; (H) CK vs. MS300.
