# Supplementary material for: Genome-wide identification and functional characterization of the Magnesium Transporter (MGT) gene family and its expression patterns to different anionic magnesium stresses in Yinshania henryi
Source: BMC Genomics. 2026 Mar 2;27:356. doi: 10.1186/s12864-026-12704-z (PMC13059214; doi:10.1186/s12864-026-12704-z)
Supplement: Supplementary file 1 — Supplementary Material 1. [file 12864_2026_12704_MOESM1_ESM.zip › Supplementary Files/Figure S6.docx]

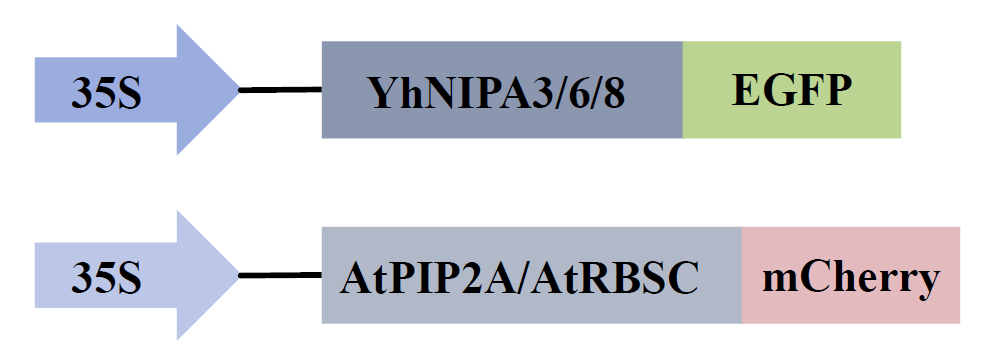


**Figure S6.** Diagram showing the construction of vectors used in subcellular localization experiments.
