# Supplementary material for: Genome-wide identification and functional characterization of the Magnesium Transporter (MGT) gene family and its expression patterns to different anionic magnesium stresses in Yinshania henryi
Source: BMC Genomics. 2026 Mar 2;27:356. doi: 10.1186/s12864-026-12704-z (PMC13059214; doi:10.1186/s12864-026-12704-z)
Supplement: Supplementary file 1 — Supplementary Material 1. [file 12864_2026_12704_MOESM1_ESM.zip › Supplementary Files/Figure S7.docx]

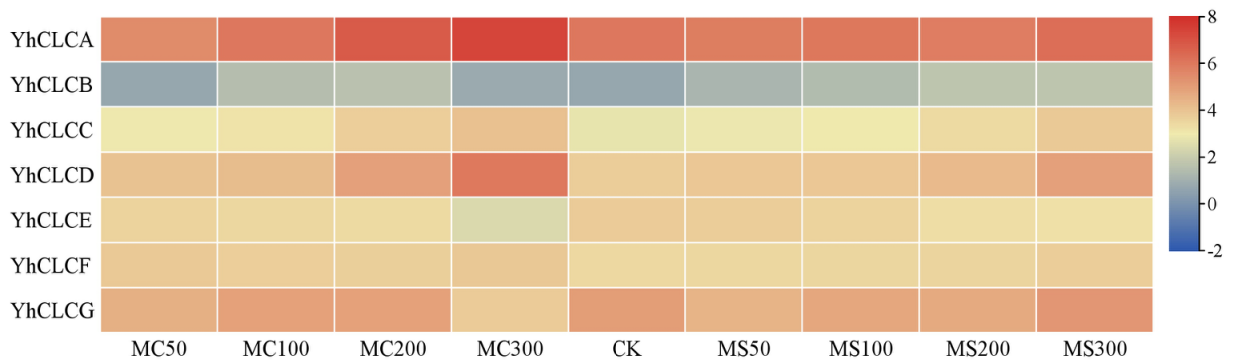


A

B


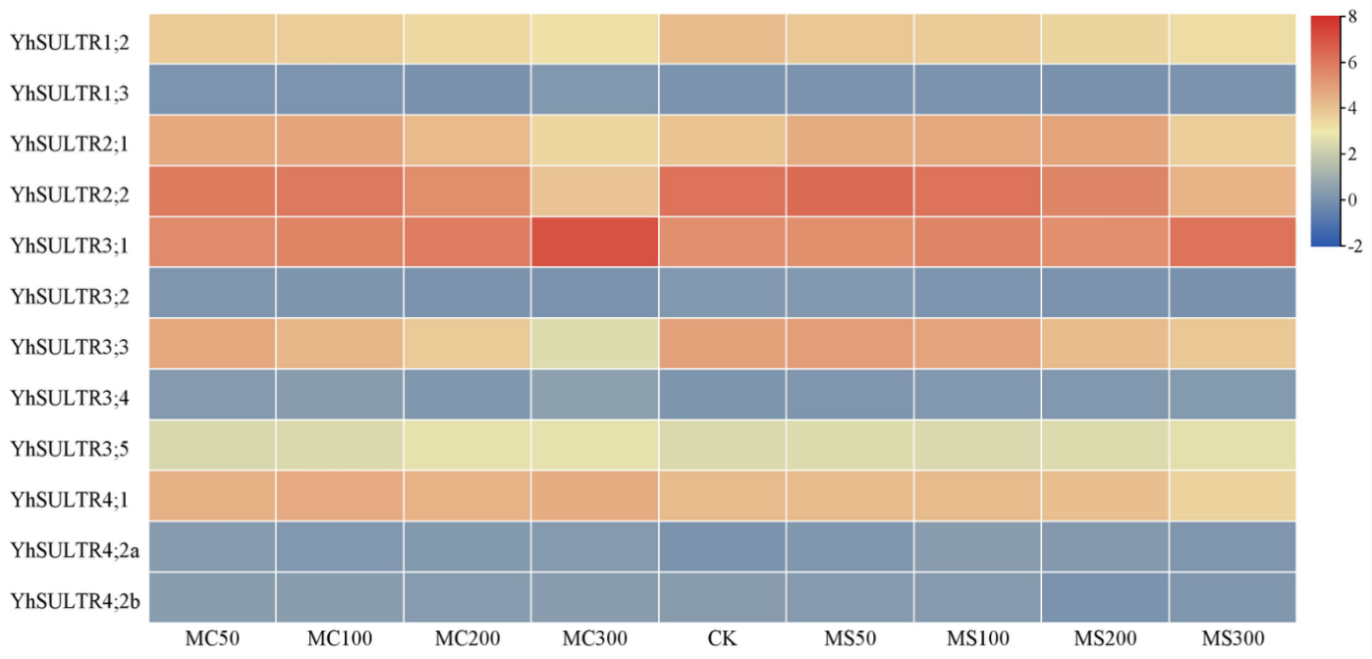


**Figure S7.** RNA-seq analysis of *YhCLC* and *YhSULTR* genes expression under MgCl₂ and MgSO₄ treatments. (A) Gene expression profiles of the *YhCLC* family genes treated with MgCl_2_ and MgSO_4_. (B) Gene expression profiles of the *YhSULTR* family genes treated with MgCl_2_ and MgSO_4_. RNA-seq data were used to determine gene expression levels. The CK (control), MC50 (50mM MgCl_2_), MC100 (100mM MgCl_2_), MC200 (200mM MgCl_2_) and MC300 (300mM MgCl_2_) represent different concentrations of MgCl_2_ treatment. MS50 (50mM MgSO_4_), MS100 (100mM MgSO_4_), MS200 (200mM MgSO_4_) and MS300 (300mM MgSO_4_) represent different concentrations of MgSO_4_ treatment. The transcript abundance levels were normalized and hierarchically clustered using log_2_(FPKM + 1) comparisons among genes across different treatments. The expression value is represented on the color scale, with red indicating high expression and blue indicating low expression.
