# Supplementary figures and images for: Genome-wide identification and functional characterization of the Magnesium Transporter (MGT) gene family and its expression patterns to different anionic magnesium stresses in Yinshania henryi
Source: BMC Genomics. 2026 Mar 2;27:356. doi: 10.1186/s12864-026-12704-z (PMC13059214; doi:10.1186/s12864-026-12704-z)

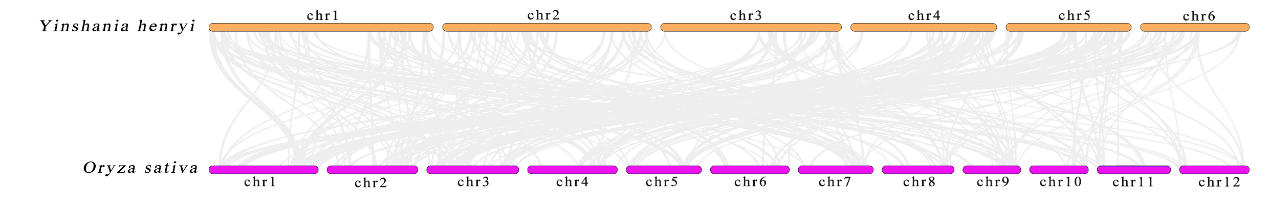


**Figure S2.** Syntenic relationship of *YhMGT* genes between *Y. henryi* and *O.sativa*.

Supplement: Supplementary file 1 — Supplementary Material 1. [file 12864_2026_12704_MOESM1_ESM.zip › Supplementary Files/Figure S2.docx]

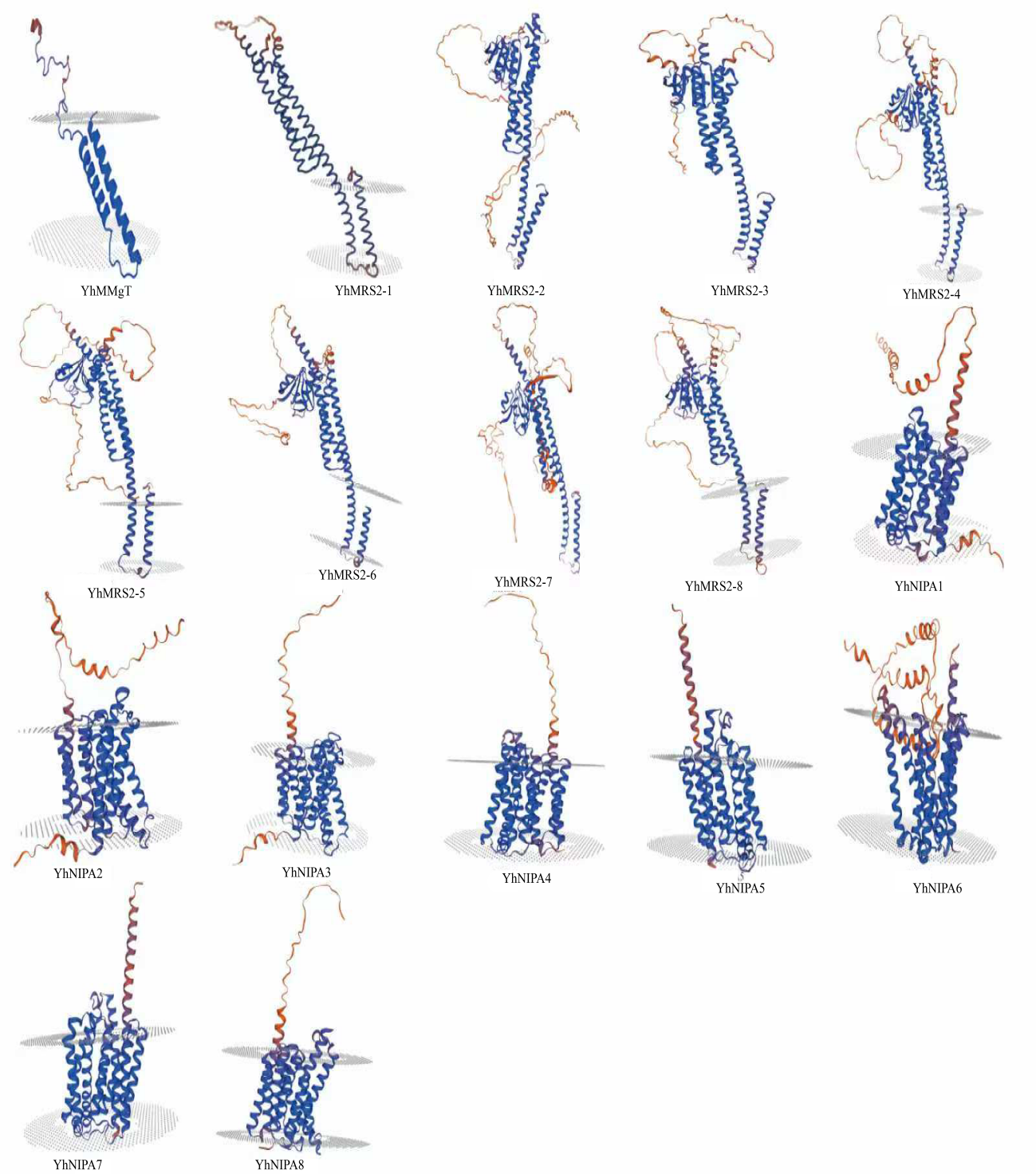


**Figure S3.** Prediction of the tertiary structure of 17 YhMGT proteins.

Supplement: Supplementary file 1 — Supplementary Material 1. [file 12864_2026_12704_MOESM1_ESM.zip › Supplementary Files/Figure S3.docx]
